# Are there physicochemical differences between allosteric and competitive ligands?

**DOI:** 10.1371/journal.pcbi.1005813

**Published:** 2017-11-10

**Authors:** Richard D. Smith, Jing Lu, Heather A. Carlson

**Affiliations:** 1 Department of Medicinal Chemistry, University of Michigan, Ann Arbor, MI, United States of America; 2 Department of Computational Medicine and Bioinformatics, University of Michigan, Ann Arbor, MI, United States of America; Danish Cancer Society Research Center, DENMARK

## Abstract

Previous studies have compared the physicochemical properties of allosteric compounds to non-allosteric compounds. Those studies have found that allosteric compounds tend to be smaller, more rigid, more hydrophobic, and more drug-like than non-allosteric compounds. However, previous studies have not properly corrected for the fact that some protein targets have much more data than other systems. This generates concern regarding the possible skew that can be introduced by the inherent bias in the available data. Hence, this study aims to determine how robust the previous findings are to the addition of newer data. This study utilizes the Allosteric Database (ASD v3.0) and ChEMBL v20 to systematically obtain large datasets of both allosteric and competitive ligands. This dataset contains 70,219 and 9,511 unique ligands for the allosteric and competitive sets, respectively. Physically relevant compound descriptors were computed to examine the differences in their chemical properties. Particular attention was given to removing redundancy in the data and normalizing across ligand diversity and varied protein targets. The resulting distributions only show that allosteric ligands tend to be more aromatic and rigid and do not confirm the increase in hydrophobicity or difference in drug-likeness. These results are robust across different normalization schemes.

## Author summary

We investigated the differences between allosteric and competitive ligands. Competitive ligands bind in the active site of a protein while allosteric ligands exhibit their effect from a remote location on the protein. Traditionally, drugs take advantage of competitive binding; however when this is not possible, an allosteric site may be a potential target. We used comprehensive, large datasets containing allosteric and competitive ligands curated from publicly available data. We carefully removed redundancy using clustering of both proteins and ligands. Here, we find allosteric ligands tend to be more rigid and aromatic than competitive ligands. However, we contradict previous studies which had indicated allosteric ligands to be more hydrophobic and drug-like. Our study provides insight into chemical traits biochemists should potential consider when designing allosteric ligands.

## Introduction

A large number of active sites have physicochemical properties that are hard to target with a drug-like small molecule [1–3]. However, these proteins can have secondary, allosteric sites with the potential to modulate function by inducing conformational or dynamic changes. Allosteric sites usually have no steric overlap with the active site. It is hypothesized that these binding sites have different physical and chemical properties which may be amenable to small molecule design when the active site has been found to be difficult to target and potentially “undruggable”.[4]

Many examples of allosterically modulated proteins have been annotated and thoroughly studied in the literature since the formalization of the theory by Monod, Wyman, and Changeux in 1965 [4,5]. Until recently, most studies have focused on characterization of allosteric ligands to a single protein [6–9]. Studies of allosteric ligands have ranged from the control of metabolic mechanisms to signal-transduction pathways [10]. Large databases such as PubChem [11], DrugBank [12], and ChEMBL [13] have allowed researchers to mine interesting patterns to help predict protein-ligand interactions. In particular, ChEMBL is annotated with descriptions of the included assays, which often note the type of interaction, including allostery [13]. An additional allosteric-specific database, the Allosteric Database (ASD), has been created with >100,000 allosteric ligands for data mining [14,15]. This study utilizes both ChEMBL and ASD to mine patterns that discriminate between allosteric and competitive ligands.

Many studies have explored allosteric mechanisms, but they tend to focus on the issue from the perspective of the protein [16–20]. Two previous studies have mined for physicochemical properties of allosteric ligands. Wang *et al*. compared the properties of the ligands contained in ASD to several databases of known biologically active compounds [21]. They showed that ligands in ASD contain more hydrophobic scaffolds and have a higher structural rigidity than the molecules in other databases, including the Available Chemicals Directory (ACD) [22], the Comprehensive Medicinal Chemistry (CMC) dataset [23], Chinese Natural Product Database (CNPD) [24], DrugBank [12,25,26], MDDR [27], and NCI Open Database [28]. In their study, ASD contained only allosteric compounds, but there was no guarantee that the other databases were free of allosteric ligands. In the second study, Van Westen *et al*. compared allosteric versus non-allosteric compounds in ChEMBL [29]. They found that allosteric compounds tended to be smaller, more lipophilic, and more rigid than non-allosteric compounds. Furthermore, they observed that allosteric ligands were not distinct from but appeared to be a subset of non-allosteric ligands. In their study, the allosteric compounds had a much narrower range of molecular weights and were more likely to adhere to Lipinski’s rule of five. Therefore, allosteric ligands were more drug-like than non-allosteric ligands.[29] They went on to focus on developing predictive models for Class B G-protein coupled receptors (GPCRs), HIV reverse transcriptase, adenosine receptors, and kinase modulators.

In our study, we focus on specifically differentiating physical properties of allosteric and competitive ligands. This asks a slightly different question than those previous studies described above. In those studies, both groups compared allosteric ligands to all other biologically active ligands as “non-allosteric ligands,” but their definitions and data compilation may have introduced allosteric complexes into the non-allosteric sets. This study uses both ASD [14,15,30] and ChEMBL [13] to have larger coverage of possible allosteric compounds than the previous studies. Furthermore, the assay descriptions in ChEMBL were used to ensure that our non-allosteric compounds are competitive inhibitors that are less likely to include allosteric compounds.

An issue that has not been properly addressed before is normalization of the data to correct for biases that come from some systems being overrepresented and having considerably more data. Many protein-ligand complexes are present multiple times in these datasets due to their importance in the field. This creates redundancy. Clearly, each protein-ligand complex should be represented only once in the datasets. In addition, some protein classes are more studied than others which creates a broader type of redundancy at both the protein level and ligand level. To address this, we performed clustering on two levels to reduce the redundancy of protein-ligand complexes and yet maintain the diversity across the sets. First, protein targets were grouped by sequence similarity, and then within each protein family, all the ligands were clustered by chemical similarity. Previous studies have only clustered molecules [21], adjusted allo and non-allo sets to be similar in size, or limited their analysis to individual protein families [29].Below, we show that allosteric ligands are more aromatic and rigid. This is in agreement with previous studies.[21,29] The previous findings that allosteric ligands are more hydrophobic or drug-like are not supported by our analysis.

## Result and discussion

Table 1 shows that the allosteric set is composed of 70,219 unique ligands which target 1048 proteins (67,749 ligands from ASD and 2470 from ChEMBL). The competitive set has 9511 unique ligands from ChEMBL, targeting 860 unique proteins. The list of SMILES strings and proteins for the allosteric and competitive ligands are given as an Excel file in the Supporting Information (allo-comp-SMILES.xlsx).

**Table 1:** The number of protein families and their protein-ligand clusters at varying cutoffs for sequence (% identity) and chemical similarity as defined by the Tanimoto coeffficient (Tc) using the extended connectivity fingerprint (ECFP6).

# Unique Ligands	#Protein Families (Providing #Protein-Ligand Clusters)			
	100%/1	90%/0.9	75%/0.75	60%/0.6
70,219 Allosteric Ligands	1048 (144,685)	923 (95,955)	858 (54,739)	759 (24,760)
9511 Competitive Ligands	860 (14,215)	757 (13,259)	681 (9157)	599 (5259)

We clustered the data at several levels to ensure that we identified robust trends that were valid for the data in fine detail and over broad categories. The clustering was done at four levels of BLAST (protein similarity) and chemical similarity: sequence identity/Tanimoto coefficient (T_c_) = 100%/≥1.0, 90%/≥0.9, 75%/≥0.75, and 60%/≥0.6. Protein clustering was performed with greedy clustering requiring the desired sequence identity in both directions (a ~ b and b ~ a). Ligands in each family were clustered on T_c_ calculated from the extended connectivity fingerprint with a diameter of six (ECFP6) using maximum dissimilarity in Pipeline Pilot [31]. These thresholds were chosen in parallel as they evenly cover a wide range of possible definitions of similarity while keeping a manageable number of clustering calculations. Polypharmacology has shown that ligands with similar chemical fingerprints have very high likelihood to bind to the same receptors [32]. Isoforms should be grouped together with 60% identity, but distinguished separately at 90% or 100% identity.

Differences in the physical properties had to be statistically significant at all four levels of clustering. The distribution of each physical property (see Table 2) was compared between the allosteric ligands and the competitive ligands, and statistically significant differences were required to have Wilcoxon p-values < 0.0001 and no overlap in the 95% confidence intervals (ci) of the medians determined from 100,000 bootstrapped samples as described in the Methods section. Below, we show that allosteric ligands are more aromatic and rigid, in agreement with previous studies.[21,29] We disagree with previous studies in terms of hydrophobicity. The difference in the median SlogP of the allosteric and competitive compounds is marginal and within the error of the SlogP method [33]. SlogP calculations have a standard deviation of 0.677 [33]. Furthermore, we find no differences in drug-like or lead-like metrics.

**Table 2:** The 29 physicochemical properties that were compared between allosteric and competitive ligands.

Category	Code	Description	Size-Corrected Property*
Atom	a_heavy (HA)	Number of heavy (non-hydrogen) atoms.	
	a_aro	Number of aromatic atoms.	a_aro/HA
	a_acc	Number of hydrogen-bond acceptor atoms.	a_acc/HA
	a_don	Number of hydrogen-bond donor atoms.	a_don/HA
	a_acid	Number of acidic atoms.	a_acid/HA
	a_base	Number of basic atoms.	a_base/HA
Bond	b_count	Number of bonds.	b_count/HA
	b_ar	Number of aromatic bonds.	b_ar/HA
	b_1rotN	Number of rotatable single bonds.	b_1rotN/HA
Physical Properties	FCharge	Total charge of the molecule.	FCharge/HA
	SlogP	Log of the octanol/water partition coefficient.	
	a_nC/HA	Number of carbon atoms (rough metric of hydrophobicity).	
	logS	Log of the aqueous solubility (mol/L).	
	Chiral	The number of chiral centers.	chiral/HA
	Rings	The number of rings.	
Drug/Lead-like	lip_druglike	One if and only if lip_violation < 2 otherwise zero.	
	lip_violation	The number of violations of Lipinski's Rule of Five.	
	opr_leadlike	One if and only if opr_violation < 2 otherwise zero.	
	opr_violation	The number of violations of Oprea's lead-like test.	

* Descriptors divided by the number of heavy atoms (HA), which corrects for the correlation between molecule size and the number of atom and bond types.

### Physicochemical differences between allosteric and competitive ligands

The medians of each physicochemical property (and their 95%ci) are given in Table 3. The values that are listed with bold font have statistically significant differences between the allosteric and competitive sets. This is determined by both a weighted Wilcoxon test (p-value <0.0001) and no overlap in the 95%ci. The physical property label is in bold font when the same statistically significant trend is seen for all levels of protein-ligand clustering.

**Table 3:** Medians (95%ci) of 29 physicochemical properties for the full dataset at all levels of clustering. Numbers in bold denote differences between allosteric and competitive compounds with p<0.0001 and no overlap in 95%ci of medians.

	60%/0.6		75%/0.75		90%/0.9		100%/1.0	
Properties	Allosteric	Competitive	Allosteric	Competitive	Allosteric	Competitive	Allosteric	Competitive
a_heavy	25 (±1)	26 (±1)	**26 (±0)**	**27 (±0)**	28 (±0)	28 (±1)	**28 (±0)**	**29 (±0)**
a_aro	12 (±0)	12 (±0)	12 (±0)	12 (±0)	**15 (±0)**	**12 (±0)**	**15 (±0)**	**12 (±0)**
**a_aro/HA**	**0.50 (±0.01)**	**0.46 (±0.01)**	**0.50 (±0.01)**	**0.462 (±0.007)**	**0.515 (±0.001)**	**0.444 (±0.005)**	**0.511 (±0.011)**	**0.429 (±0.006)**
a_acc	**3 (±0)**	**4 (±0)**	4 (±0)	4 (±0)	4 (±0)	4 (±0)	4 (±0)	4 (±0)
**a_acc/HA**	**0.136 (<0.001)**	**0.148 (±0.005)**	**0.136 (±0.001)**	**0.143 (<0.001)**	**0.138 (±0.001)**	**0.143 (<0.001)**	**0.136 (<0.001)**	**0.143 (±0.004)**
**a_don**	**1 (±0)**	**2 (±0)**	**1 (±0)**	**2 (±0)**	**1 (±0)**	**2 (±0)**	**1 (±0)**	**2 (±0)**
**a_don/HA**	**0.045 (<0.001)**	**0.083 (±0.003)**	**0.042 (<0.001)**	**0.077 (±0.003)**	**0.038 (<0.001)**	**0.074 (±0.003)**	**0.037 (<0.001)**	**0.076 (±0.002)**
a_acid	0 (±0)	0 (±0)	0 (±0)	0 (±0)	0 (±0)	0 (±0)	0 (±0)	0 (±0)
a_acid/HA	0 (±0)	0 (±0)	0 (±0)	0 (±0)	0 (±0)	0 (±0)	0 (±0)	0 (±0)
a_base	0 (±0)	0 (±0)	0 (±0)	0 (±0)	0 (±0)	0 (±0)	0 (±0)	0 (±0)
a_base/HA	0 (±0)	0 (±0)	0 (±0)	0 (±0)	0 (±0)	0 (±0)	0 (±0)	0 (±0)
**b_count**	**44 (±1)**	**49 (±1)**	**47 (±0)**	**52 (±1)**	**51 (±1)**	**55 (±1)**	**51 (±0)**	**56 (±0)**
**b_count/HA**	**1.808 (±0.008)**	**1.92 (±0.01)**	**1.800 (<0.001)**	**1.932 (±0.007)**	**1.800 (±0.006)**	**1.943 (±0.005)**	**1.813 (±0.002)**	**1.951 (±0.006)**
b_ar	12 (±0)	12 (±0)	12 (±0)	12 (±0)	**16 (±0)**	**12 (±0)**	**16 (±0)**	**12 (±0)**
**b_ar/HA**	**0.500 (<0.001)**	**0.462 (±0.005)**	**0.516 (±0.006)**	**0.462 (±0.009)**	**0.522 (±0.006)**	**0.444 (±0.009)**	**0.515 (<0.001)**	**0.44 (±0.01)**
**b_1rotN**	**4 (±0)**	**5 (±0)**	**4 (±0)**	**5 (±0)**	**5 (±0)**	**6 (±0)**	**5 (±0)**	**6 (±0)**
**b_1rotN/HA**	**0.167 (<0.001)**	**0.185 (±0.003)**	**0.167 (<0.001)**	**0.188 (±0.003)**	**0.172 (<0.001)**	**0.195 (±0.005)**	**0.174 (<0.001)**	**0.2 (±0)**
FCharge	0 (±0)	0 (±0)	0 (±0)	0 (±0)	0 (±0)	0 (±0)	0 (±0)	0 (±0)
FCharge/HA	0 (±0)	0 (±0)	0 (±0)	0 (±0)	0 (±0)	0 (±0)	0 (±0)	0 (±0)
**SlogP**	**3.42 (±0.03)**	**3.26 (±0.07)**	**3.61 (±0.02)**	**3.52 (±0.05)**	**3.85 (±0.01)**	**3.62 (±0.03)**	**3.89 (±0.01)**	**3.59 (±0.03)**
**a_nC/HA**	**0.731 (±0.001)**	**0.741 (±0.004)**	**0.731 (<0.001)**	**0.750 (±0.003)**	**0.731 (±0.001)**	**0.750 (±0.006)**	**0.731 (<0.001)**	**0.744 (±0.006)**
logS	**-4.48 (±0.03)**	**-4.61 (±0.08)**	**-4.79 (±0.02)**	**-4.87 (±0.05)**	-5.13 (±0.01)	-5.13 (±0.04)	-5.15 (±0.01)	-5.15 (±0.04)
chiral	0 (±0)	0 (±1)	**0 (±0)**	**1 (±0)**	**0 (±0)**	**1 (±0)**	**0 (±0)**	**1 (±0)**
chiral/HA	0 (±0)	0.00 (±0.02)	**0 (±0)**	**0.029 (±0.002)**	**0 (±0)**	**0.032 (±0.001)**	**0 (±0)**	**0.033 (±0.001)**
rings	3 (±0)	3 (±0)	3 (±0)	3 (±0)	4 (±0)	3 (±1)	4 (±0)	3 (±1)
lip_druglike	1 (±0)	1 (±0)	1 (±0)	1 (±0)	1 (±0)	1 (±0)	1 (±0)	1 (±0)
lip_violation	0 (±0)	0 (±0)	0 (±0)	0 (±0)	0 (±0)	0 (±0)	0 (±0)	0 (±0)
opr_leadlike	1 (±0)	1 (±0)	1 (±0)	1 (±0)	1 (±0)	1 (±0)	1 (±0)	1 (±0)
opr_violation	0 (±0)	1 (±1)	**0 (±0)**	**1 (±0)**	1 (±0)	1 (±0)	1 (±0)	1 (±0)

#### Allosteric ligands are more aromatic and constrained

**Fig 1:**
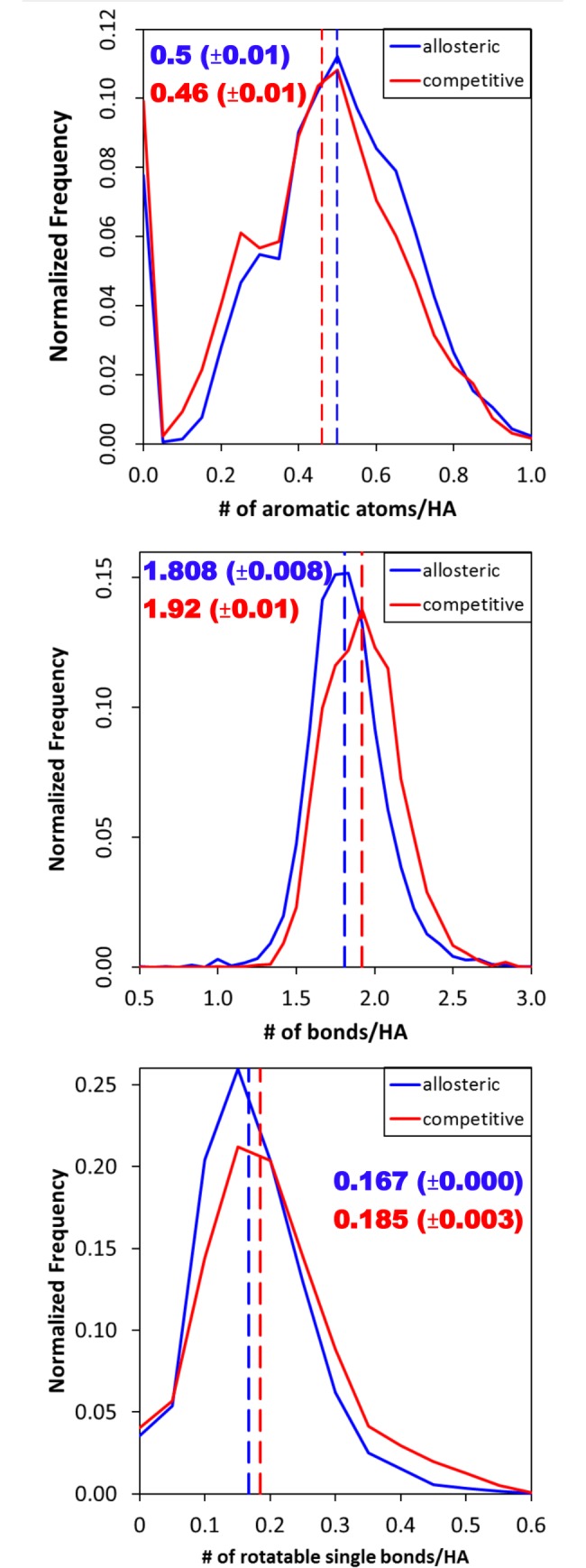
The normalized histograms of the number of aromatic atoms corrected by size, the number of bonds per heavy atom (HA), and the number of rotatable single bonds per HA for the full dataset (60%/0.6 clustering). The median (dashed line) is labeled on the graph with its 95%ci derived from bootstrap sampling.

We find that there are multiple descriptors that indicate the allosteric ligands are more rigid. The distributions show that allosteric ligands have more aromatic atoms and fewer bonds per heavy atom (meaning fewer saturated bonds). Furthermore, there are fewer rotatable single bonds per heavy atom (HA). The distributions are shown in Fig 1.

The combination of the increase in aromatic atoms and the decrease in the number of rotatable single bonds in the allosteric ligands suggests that these molecules tend to be more rigid. This rigidity is somewhat surprising since allosteric binding sites frequently undergo a change in conformation upon ligand binding. The change of protein flexibility upon ligand binding has been seen by Demerdash *et al*. [17]. That study used a Support Vector Machine (SVM) approach and indicated that the deformation energy and change in solvent-accessible surface area (SASA) of the protein residues are important features in predicting an allosteric hotspot. The SVM models indicated that allosteric hotspots would form dense networks within the protein. They did not look specifically at the residues in contact with allosteric ligands, and they make no comment on their flexibility [17]. Panjkovich and Daura also noted that a large change in B-factors can be used to indicate the location of allosteric binding sites [20]. However, a recent study by Li *et al*. suggested that the pocket flexibility (normalized B-factor) and pocket depth are not significantly different for allosteric binding sites.[18] Although, they implied from these factors that arthosteric and orthosteric sites would have similar flexibility, they state that the allosteric sites are “buried and more compact”.

The decrease in flexibility of allosteric compounds is consistent with two other studies on large datasets of allosteric compounds[21,29]. In the work of Wang *et al*, allosteric ligands have significantly fewer rotatable bonds than drug molecules from DrugBank (their criteria was p-value <0.01, two sample t-test) [21]. Van Westen *et al*. describes that allosteric ligands have more sp2 hybridized carbons, fewer sp3 hybridized carbons, and more aromatic bonds [29] compared to non-allosteric compounds targeting transmembrane proteins. Taken together, it appears that relatively rigid ligands bind to allosteric sites, inducing a change in flexibility of the protein as it adapts to the presence of the allosteric ligand.

#### Allosteric ligands are not necessarily more hydrophobic

The number of hydrogen-bond donors and acceptors per HA (a_don/HA and a_acc/HA) indicate that allosteric ligands have fewer hydrophilic functional groups. Also, differences in SlogP are statistically significant with the weighted Wilcoxon test and when comparing the 95% confidence intervals, but there is only a difference of 0.2 log units in the medians. This is within the error of the SlogP calculations [33], and therefore, we conclude the difference is negligible, regardless of statistical analysis.

We were surprised to find this result because previous studies have indicated that allosteric ligands are more hydrophobic and have hydrophobic binding sites. A study by Li *et al*. [18] found allosteric binding sites contain more hydrophobic surface area, and are likely to bind more hydrophobic ligands. Hydrophobicity is also used as an important characteristic for the prediction of allosteric sites by Huang *et al*. [34] and by Demerdash *et al*.[17] Although these latter two studies did not directly implicate hydrophobicity, many of the characteristics implicated by the models, such as hydrogen-bonding characteristics of the active site do. Hydrophobicity was also important in the prediction of allosteric protein-ligand interactions using models developed by Li *et al*.[35] In that study the hydrophobicity, as determined by a hydrophobic matching algorithm, had the highest contribution to Alloscore, which is their metric to predict allosteric interactions. The Monod-Wyman- Changeux model also states that protein-protein or subunit interfaces are frequent allosteric binding sites [4], and protein-protein interfaces have generally been shown to be more hydrophobic in nature.[36–38]

The structural properties of allosteric binding sites likely constrain the chemical characteristics of allosteric ligands. Studies for identifying allosteric binding sites show that the SASA [17], the number of hydrogen bonds [17], interaction between residues, local hydrophobic density [18,34], pocket size [17,34], and correlated features are important for describing an allosteric binding site. Here, we investigate the hydrophobicity of the lignds (SlogP/logS) which does not necessarily represent to the hydrophobicity of the binding site. Van Westen *et al* observed that allosteric compounds for GPCRs tend to be more lipophilic (higher logP), more rigid (higher sp2 C and lower sp3 C), and relatively smaller than non-allosteric ligands from ChEMBL data [29]. It is difficult to determine the overlap between our dataset and van Westen’s as their compounds were derived from ChEMBL and were not provided with their publication.

Wang *et al*. compared allosteric ligands from ASD with compounds from databases like DrugBank, MDDR, ACD, etc [21]. However, the compounds in these databases can have many mechanisms to bind target proteins, which are not necessarily binding at the active site. Even so, they also showed that the allosteric ligands contain more hydrophobic scaffolds and are more rigid. Wang *et al*. determined that SlogP and Total Polar Surface Area (TPSA) of allosteric ligands were statistically significant from six other compound libraries. Although statistically significant, the distributions displayed do not show a large difference and have large error bars. It should be noted that they utilized an older version of the ASD database, which has shown a large expansion since the 2010 version (7,851 compounds compared to 67,749).

As a sanity check, we also performed single-level clustering on the ligands (ligands were simply clustered by chemistry without regard for protein families). The competitive ligands were grouped together and the allosteric ligands were grouped together, and then each set was clustered at T_c_ of 0.6, 0.75, 0.9, and 1.0 and compared to see if the above trends held. The differences between the two sets at each clustering level can be seen in Supporting Information (S1 Table). When compared to Table 3 the conclusion that allosteric ligands were more aromatic and constrained can still be drawn. Only one variable, the number of single, rotatable bonds was not significant at all levels, but the number of single, rotatable bonds per HA remained significant. SlogP is marginally different between the two ligand sets, with no significant difference in logS, hence allosteric ligands do not appear to be more hydrophobic, as seen with the two-level clustering.

**Fig 2:**
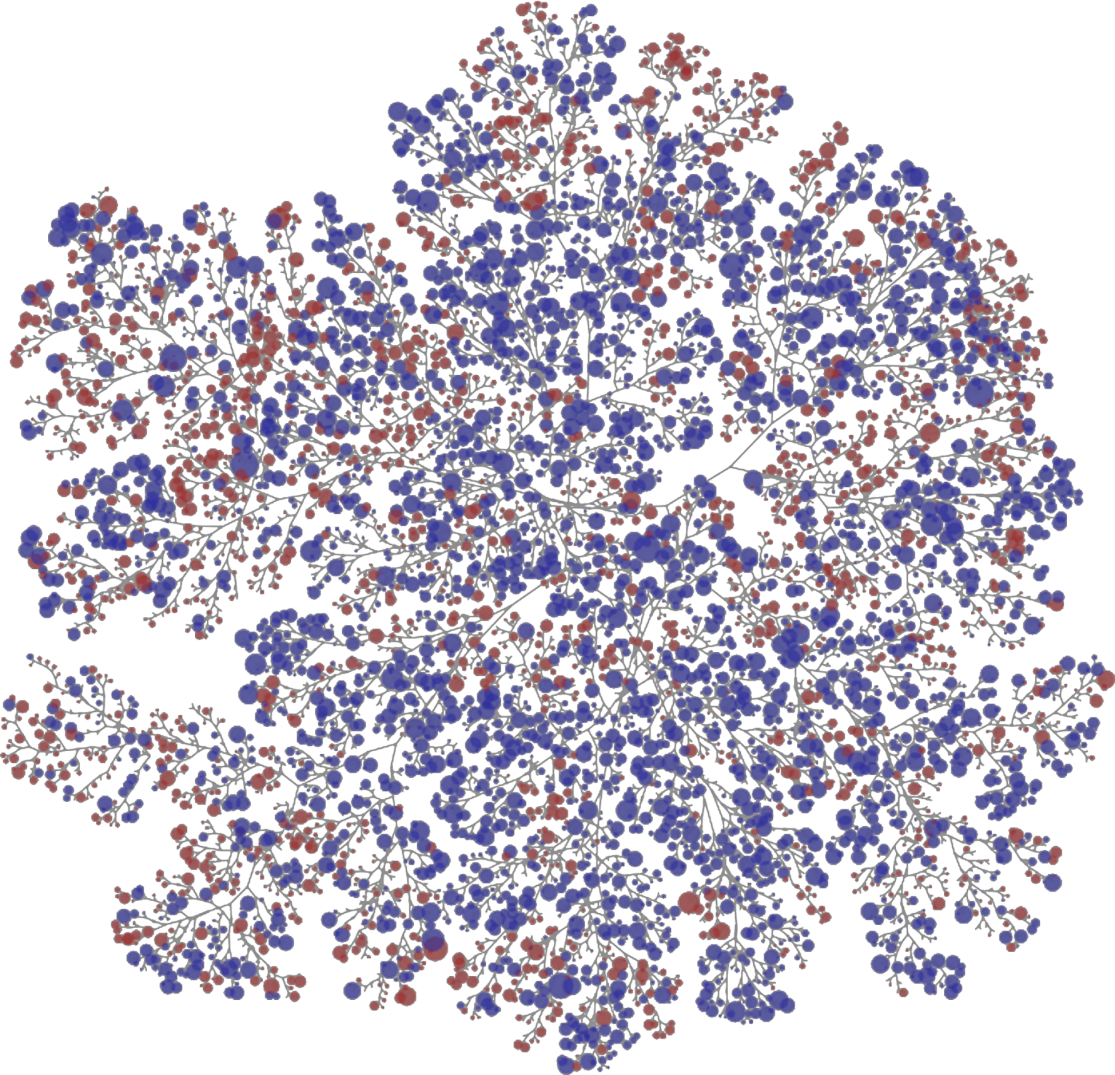
ChemTreeMap [39] of the allosteric (blue) and competitive (red) ligands is a hierarchical tree based on grouping ligands by the Tc of the ECFP6 fingerprints. The size of each circle represents the number of ligands in a cluster of Tc ≥ 0.6. Though a few small regions of chemical space are dominated by one set or the other, the large number of branches with interdigitated red and blue circles shows that there is a great deal of chemical similarity between the allosteric and competitive ligands. To quantify the overlap, we should note that 623 out of 70,219 allosteric ligands (1%) are within Tc ≥ 0.6 of a competitive ligand, and 582 of the 9511 competitive ligands (6%) are within Tc ≥ 0.6 of an allosteric compound.

**Fig 3:**
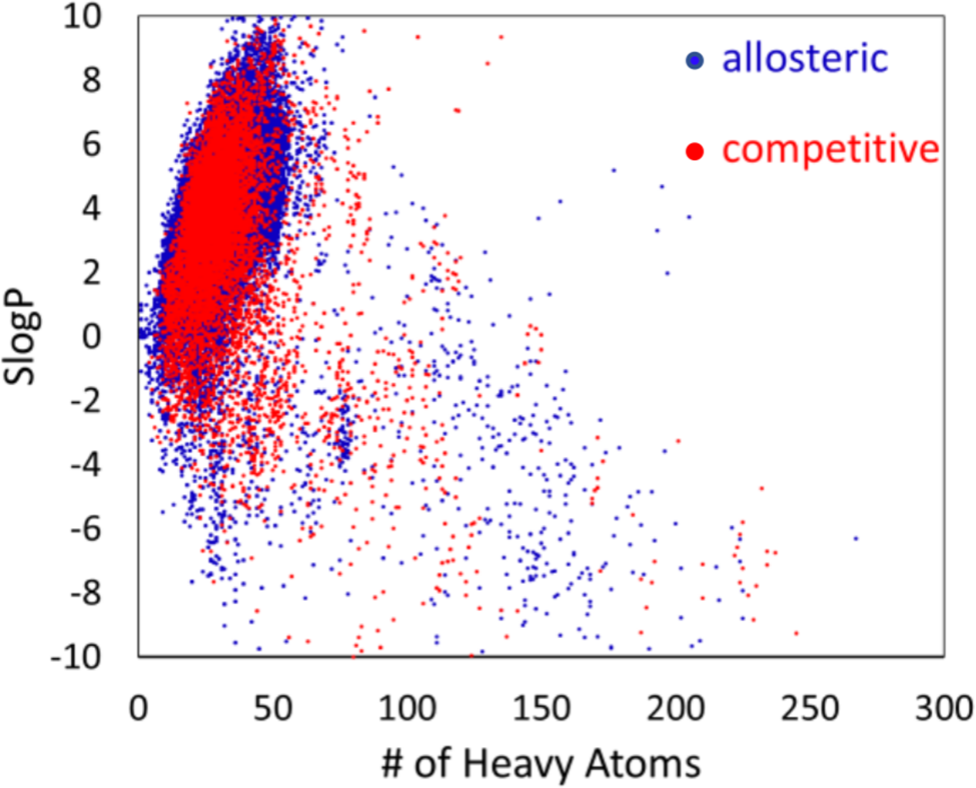
SlogP versus the number of heavy atoms. Clustering was performed at 100% sequence identity and Tc = 1.0.

Overall, the allosteric and competitive ligands span similar chemical space. Fig 2 shows a map of the chemical similarity based on ECFP6; the fingerprint was used to give a broader characterization of similarity rather than focusing on one property. The map was generated by ChemTreeMap which was developed in our lab [39]. Van Westen *et al*. suggested that allosteric modulators form a subset of non-allosteric modulators, based on the observation that their allosteric ligands had a narrower range of molecular weight and covered a smaller area in a scatter plot of logP vs molecular weight. However, when we compare our allosteric and competitive sets based on ligand similarity (T_c_), the compounds of both categories appear to cover similar chemical space in Fig 2. Furthermore, Fig 3 gives a graph of SlogP vs HA for our data that shows both competitive ligands and allosteric ligands span the same range of chemical features.

### Distributions of protein targets

**Fig 4:**
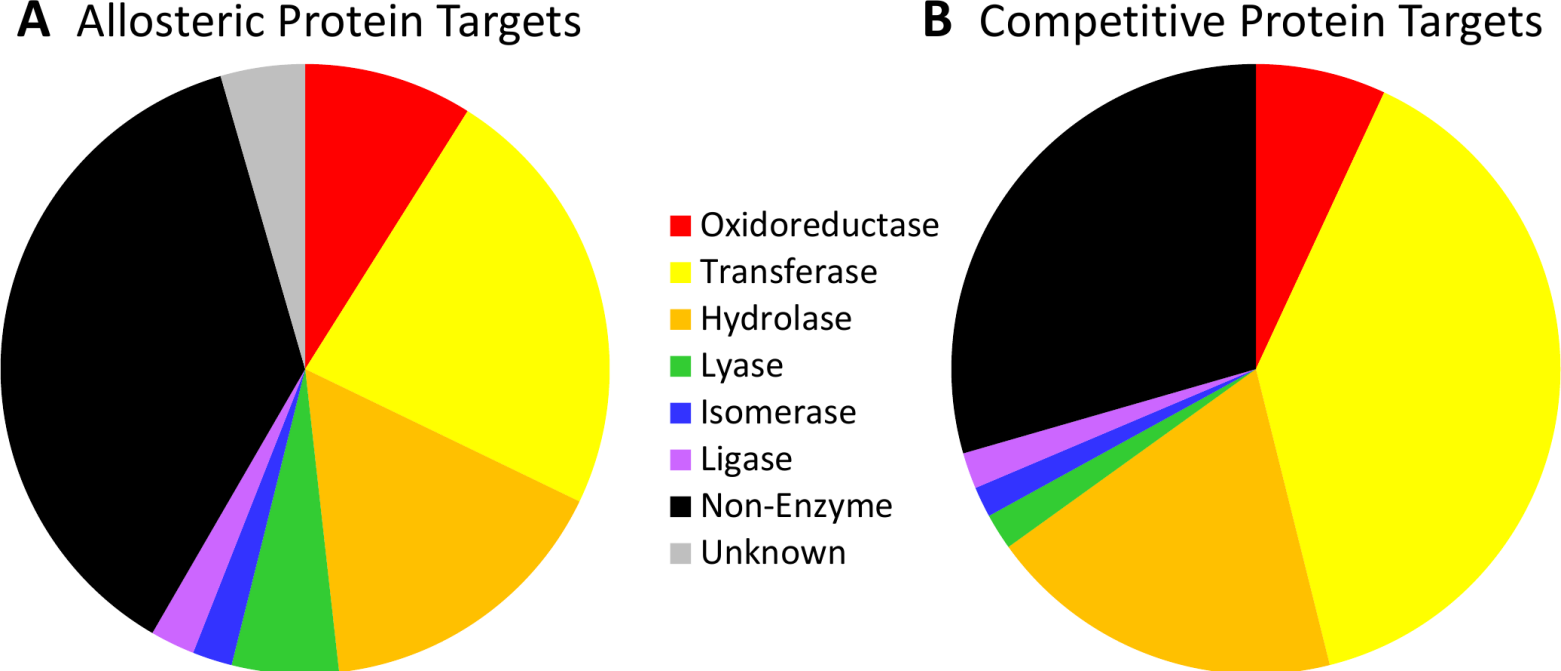
The distribution of protein targets for the allosteric (A) and competitive (B) compounds.

Due to the different modes of action, one might expect to see a difference in the types of proteins targeted by allosteric vs competitive ligands as this depends upon protein function (Fig 4). One might also expect that the range of proteins could contribute to any differences in our findings versus previous studies.

When clustering the dataset at 60%, the number of unique protein families in the full allosteric dataset was 759, while there were 599 different protein families in the competitive dataset. ASD 3.0 has increased by >400% since the initial ASD 1.0, due to the significant expansion of allosteric drug discovery [14,15,30]. Three categories of allosteric proteins are dramatically augmented from ASD 1.0 to ASD 3.0: kinases (from 46 to 207), GPCRs (from 48 to 118), and ion channels (from 21 to 134), which are highly associated with the therapeutic targets in recent drug discovery studies [12,40]. As one would expect, there are differences in the protein targets of allosteric and competitive ligands. A large portion (46.3%) of allosteric ligands target GPCR proteins, while 29.1% of competitive compounds bind to these proteins. A larger percentage of allosteric compounds (14.4%) target neuronal proteins (for example, the GABA receptor, glutamate receptor, and acetylcholine receptor) compared to competitive compounds (8.1%). Li *et al*. found that the majority of allosteric proteins obtained from ASD were transferases (44.8%) instead of GPCRs. This percentage is artificially high as it is only the percentage of enzymes, not all proteins, since they did not have a category designation for non-enzymatic proteins.[18] In our dataset, 21.9% of allosteric ligands and 13.2% of competitive ligands target transferases. For enzymes in the datasets, 53.3% of the targets in the allosteric set and 26.7% of the targets of the competitive set are transferase. A complete list of the protein targets in each set is given in the Supporting Information (S2 Table).

A large number of GPCR-based allosteric compounds appear in ASD v3.0 [30]. GPCRs play a critical role in multiple diseases, and there are significant efforts in developing new GPCR-based drugs [41]. Moreover, many subtype GPCRs have high sequence similarity in their orthosteric sites. It has been difficult to obtain high selectivity when targeting those sites. In recent years, targeting allosteric sites has been a major thrust for developing GPCR drugs [42,43].

Van Westen *et al*. built a dataset from ChEMBL (417 targets for allosteric ligands and 1,869 for non-allosteric ligands) and examined the distribution of targets and found a bias to transmembrane proteins (~50%). That study did not remove redundancy on the protein level or the ligand level. Our study reduced this bias caused by overrepresented proteins and ligands utilizing two-level clustering for both protein and ligands to remove redundancy (see Methods).

### Protein-ligand clusters

One reason that we may have some disagreement with previous findings regarding hydrophobicity of allosteric ligands is that our sets of compounds are different from those earlier results. The number of protein-ligand clusters is different for each protein and is dictated for each protein by the chemical diversity of its ligands. In our full dataset, there are 70,219 allosteric ligands and 9511 competitive ligands. These are spread across 1048 and 860 protein targets, respectively. Some compounds can interact with more than one target, and the number of protein-ligand clusters ranges from 599 clusters for the competitive compounds at 60/0.6 clustering up to 144,685 for allosteric ligands clustered at 100/1.0 (Table 1). The clusters are used to reduce the bias from heavily studied proteins vs newer targets.

The number of ChEMBL ligands in our datasets is smaller than those used by van Westen *et al*. because our datasets were built with compounds from experiments that expressly defined assay activity as either allosteric or competitive. Therefore, evidence was identified using restrictive search terms on the assay descriptions as opposed to parsing the language of the ChEMBL documents. The allosteric set from ChEMBL is smaller (our 2,470 vs. 17,829 in van Westen *et al*.) due to the fact only “alloster*” was used in the keyword search of the *assay description* and a manual curation was performed to remove high throughput screening (HTS) data. However, van Westen *et al*. used several additional terms, which may imply allostery, when searching the whole *document* from ChEMBL. Discarding the HTS data also limits the size of the dataset used in this study. Using the assay description and the more restrictive search term helps to ensure that each ligand obtained from ChEMBL is indeed an allosteric modulator. Our “competitive” dataset obtained from ChEMBL is also smaller than their “non-allosteric” set, since the focus is on competitive inhibitors annotated in the assay descriptions and ambiguous mechanisms are not included in our set. The growth of allosteric ligands in ASD brings the dataset to a size comparable to van Westen’s study.

### Conclusions

This study compares common features of allosteric ligands to competitive ligands in order to understand their unique chemical properties. The datasets were carefully curated to ensure the correct designation of their known mechanisms. Verifying the assays assures that we are only comparing allosteric ligands to competitive ligands. Also, this study provides a dataset as large as or larger than previous studies performed on allosteric ligands.

We took great care in normalizing the data so that frequently studied proteins did not overly bias the outcomes. The results indicate that allosteric compounds tend to be more aromatic and rigid. This is supported by allosteric ligands having more aromatic atoms per heavy atom. It is also supported by a decrease in chemical saturation and fewer rotatable single bonds for allosteric ligands. The rigid nature of these ligands, combined with other studies that have shown protein allosteric hotspots are more flexible, suggest that the protein may adapt its conformation to the more rigid ligand and inducing an allosteric conformational change.

This also has application in the drug discovery process. Knowledge of the physical properties of allosteric ligands, may allow researchers in drug discovery understand if a particular molecule or drug candidate would have a higher potential to bind allosterically. Investigating all allosteric ligands, we have found them to be more rigid and aromatic in nature. This may assist in determining a mode of action, when one is not known. Additionally, if the known binding site of a protein is difficult to target, one might be able to limit or enrich their search to molecules which are rigid and more aromatic in order to increase likelihood of identifying an allosteric site, which one might target instead.

## Methods

### Ethics statement

No humans or animals were used in this research.

### Data collection

The information on ligands (SMILES strings) and their target proteins (FASTA sequences) were collected from ASD version 3.0 [30] and ChEMBL version 20 [13,44]. Filtering and data analysis was performed as follows.

The allosteric data was extracted from both ChEMBL and ASD. ASD data is imported without filtering since it focuses exclusively on allosteric mechanisms. Data from ChEMBL was filtered to select appropriate allosteric ligands to augment the ASD dataset. The entire set of ChEMBL assay descriptions (‘description’ field in the assays table of the ChEMBL MySQL database) was filtered for those which contain the term “alloster*” (the “*” indicates a wild card which allows any set of characters to follow the search pattern). All ligands which were characterized by HTS assays were then removed due to the high error rate in HTS approaches. Allosteric-relevant assays from the remaining set were then selected by manually reading the descriptions. Only active molecules were kept based on the reporting of a non-zero ‘standard value’ field in the ‘activities’ table of the ChEMBL MySQL database. The dataset for competitive compounds, which is based only on ChEMBL, was obtained by searching for the term “compet*”. The dataset was also filtered using by-hand verification of assay descriptions. For completeness, the list of ChEMBL assays is provided in the Supporting Information (allo-comp_ChEMBL_assays.xlsx). We should note that we did not include binding affinities or inhibition constants in our analysis because some ligands did not have explicit K_d_, K_i_, or IC_50_ data given.

#### Removing seven overrepresented molecules

**Fig 5:**
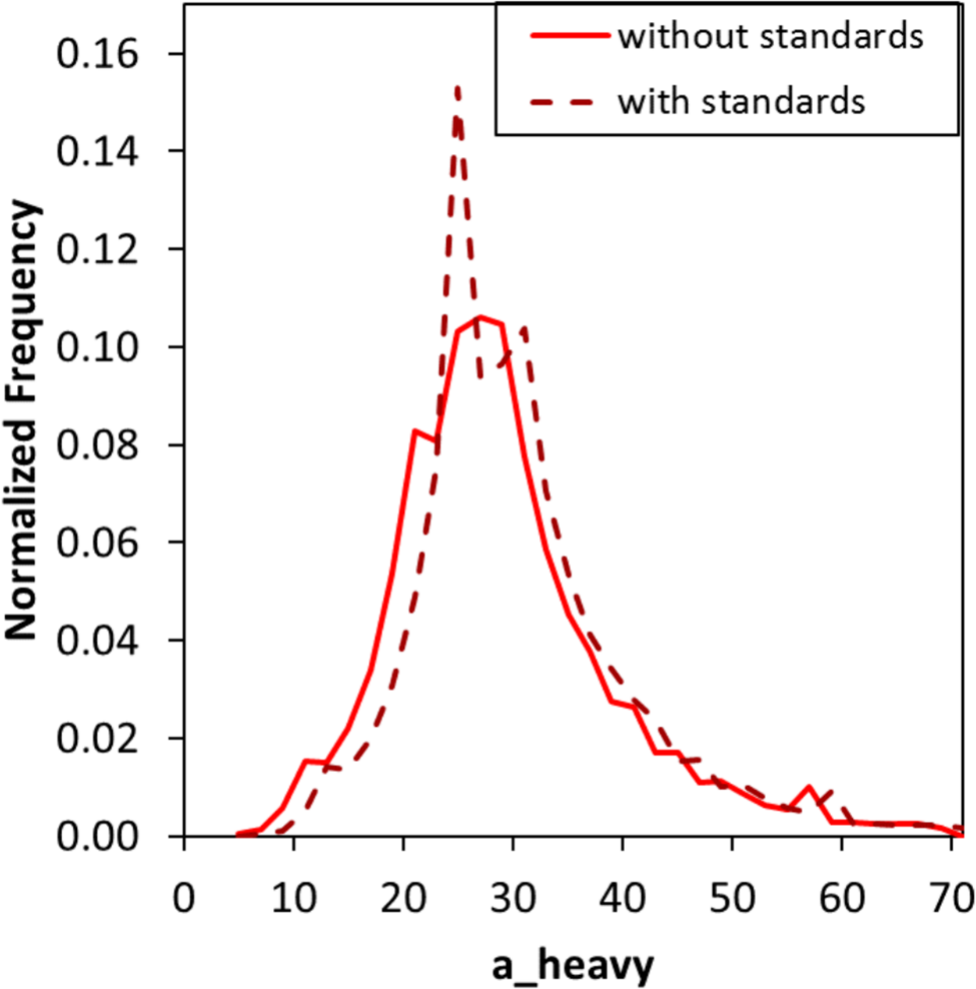
The normalized distribution of heavy atoms for the ligands in the competitive set, with (brown, dashed line) and without (red, solid line) the seven kinase standards.

There are seven compounds in the competitive set that are overrepresented because of a single study where each was tested against a panel of >200 kinases. ChEMBL has included all data and appropriately marked them as active [45]. Fig 5 shows the influence that these seven assay standards have on the set of thousands of competitive ligands; clearly, they had to be removed because they reflect a man-made artifact.

### Calculation of compound descriptors

Molecular Operating Environment (MOE) 2014 [46] was used to calculate the compound descriptors. The SMILES were converted to molecular structures in MOE, and ligands were properly protonated using the default options of the *wash* procedure in the MOE database viewer. The *wash* procedure ionizes strong bases and strong acids at a pH of 7 and adds explicit hydrogens to each molecule.

Physicochemical descriptors were calculated to characterize the molecules, including atom counts, bond counts, physical properties (SlogP, FCharge), and drug/lead-like characteristics. Some descriptors are highly correlated with ligand size; for example, the number of bonds is highly correlated to the number of heavy atoms (HA). Therefore, descriptors were also examined when corrected for size by dividing by the number of heavy atoms (e.g. chiral/HA, a_nC/HA). All descriptors available in MOE were computed, but we only examined descriptors that are experimentally measurable and can be predicatively modified, meaning chemical substitutions can logically alter the descriptor. The list of the descriptors we analyzed is in Table 2.

### Removing redundancy

**Fig 6:**
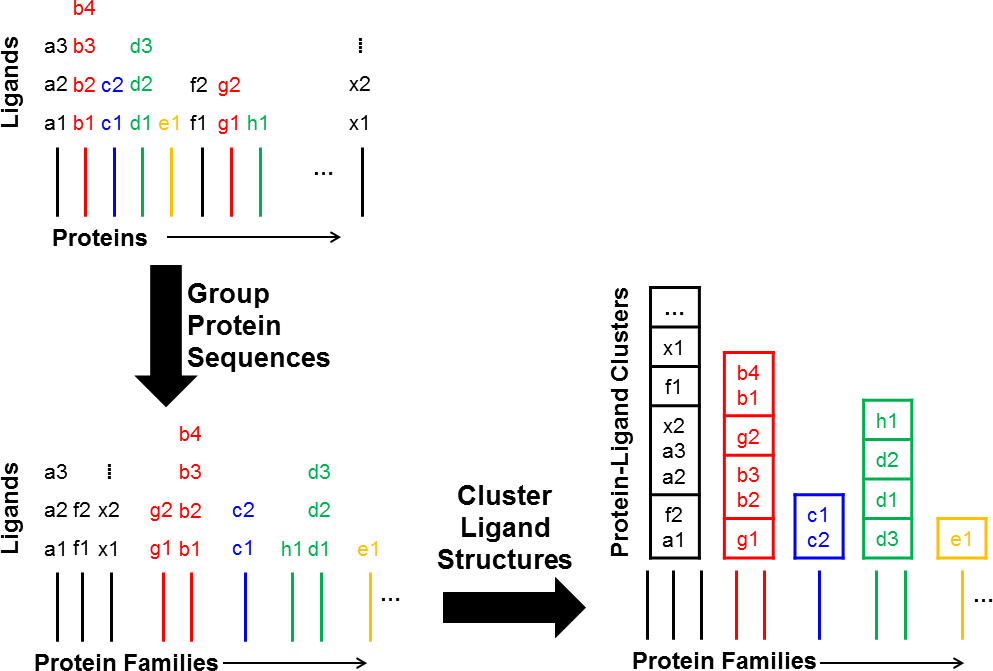
Clustering the data in two levels. The horizontal axis represents “protein space.” In the first step, similar proteins are grouped into families. The vertical axis represents “ligand space.” The second step clusters all the ligands associated with each protein in the protein family. The final boxes are the protein-ligand clusters used in normalizing the data.

Any identical data (exact same protein with exact same ligand) was culled to one entry. Then, a two-level clustering method was used to remove redundancy. The target proteins were grouped by sequence identity with BLAST [47] (x-axes of Fig 6) by running *formatdb* and *blastp* on the protein sequences with default parameters. Four different thresholds for sequence identity were used (60%, 75%, 90%, and 100%) to group homologous sequences into protein families. All ligands for the proteins in each family are then clustered by Pipeline Pilot 9.2 [31] (final boxes for ligands in Fig 6) with the *Cluster Molecules* component using the maximum dissimilarity [48] setting. The ligand similarity is quantified by the Tanimoto coefficient (T_c_) based on the ECFP6 fingerprints [49]. Four different thresholds for similarity were used to cluster the ligands (T_c_ = 0.6 for protein families at 60% sequence identity, 0.75 for 75% sequence identity, 0.9 for 90% sequence identity, and 1 for 100% sequence identity). To be considered robust, we required any statistically significant difference in physical properties to be present at all four levels of clustering.

**Fig 7:**
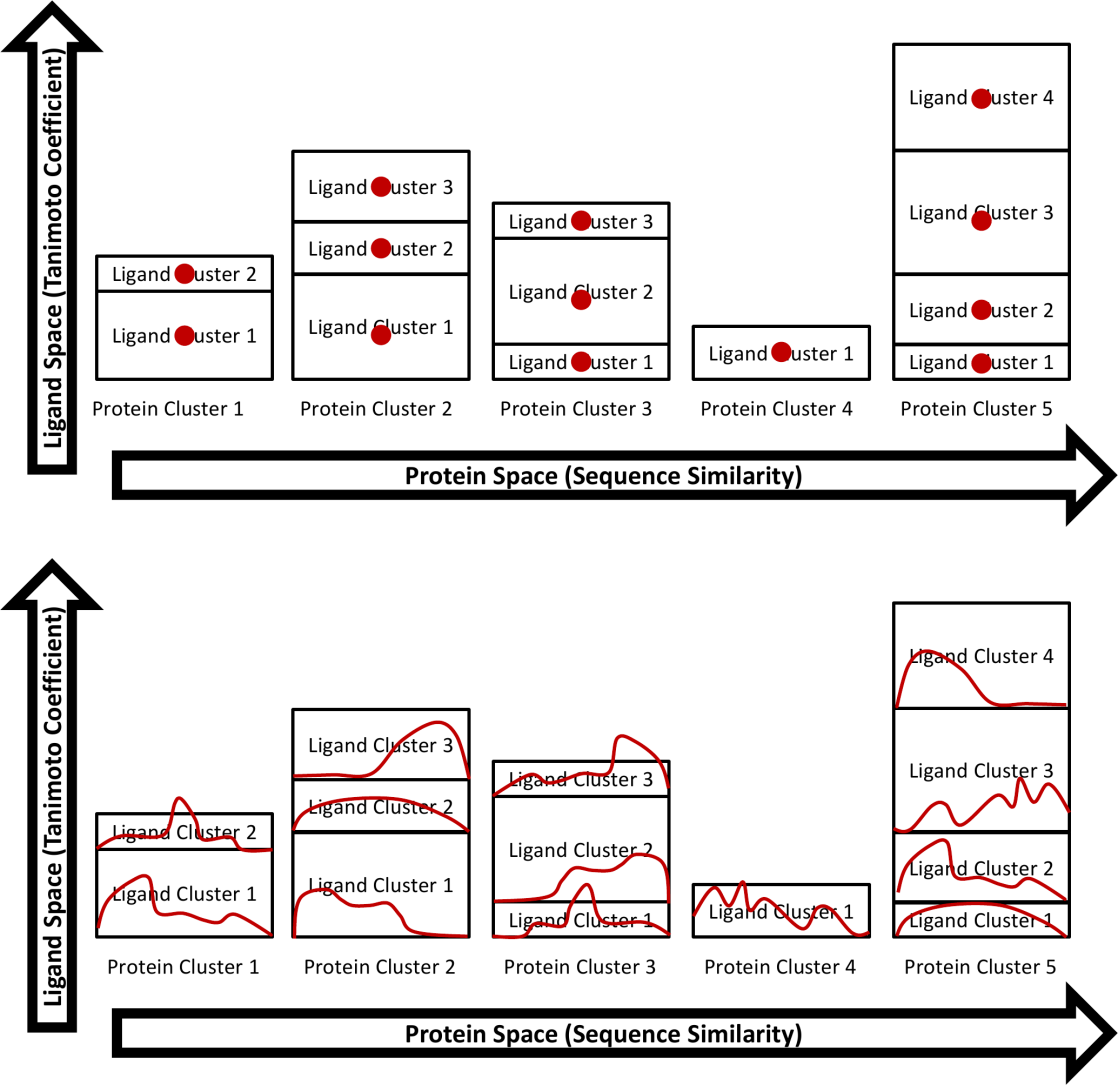
Weighting the data. In the top scheme, the center of each protein-ligand cluster is chosen, and only the centers are used to calculate the distribution of properties. In the bottom scheme, all ligands of each cluster contribute to the cluster’s normalized distribution of physical properties. The normalized distributions compensate for the different number of ligands in each cluster. The normalized distributions of all clusters are simply added to give the distribution of physical properties for the entire set of data.

This process allowed us to examine the data in both fine detail and across broader levels. The resulting dataset is described in Table 1. One method of normalizing for the different sizes of each protein-ligand cluster is to choose the “center” of each cluster to represent those molecules in the data analysis. The center is defined as the molecule with smallest sum of T_c_ distances to other molecules in the cluster (top scheme in Fig 7). The centers of each cluster were used to calculate the distribution of physical properties. All results based on the analysis of the centers are given in the Supporting Information for completeness (S3 Table). An alternative method of calculating these distributions (bottom scheme of Fig 7) is described in the next section, and it is the focus of the analysis in the results and discussion. However, the results from both normalization methods are nearly identical.

### Weighting to remove redundancy

Traditional clustering chooses only the center for each cluster. Unfortunately, this loses the information from the other ligands in the cluster. To include every molecule in the analysis, a simple adjustment was needed to correct for the different number of ligands in each protein-ligand cluster. We used the normalized distributions so that protein-ligand clusters of different sizes had the same contribution to the combined analysis (all histograms sum to 1.0). This is shown in the bottom scheme of Fig 7. The normalized distributions from each cluster are added to calculate the mean and median of the entire set. For the histograms, binning was done at intervals of 1 for discrete variables and 0.001 for continuous variables.

### Data analysis to identify statistically significant differences in physicochemical properties of allosteric and competitive ligands

#### Wilcoxon

The differences between the allosteric and competitive distributions were assessed by two methods: the Wilcoxon rank-sum test [50] for data from the centers of the clusters and the weighted Wilcoxon rank-sum test for our normalized distributions. Many descriptors have non-Gaussian distributions; therefore, the non-parametric Wilcoxon test was the appropriate choice. The weighted Wilcoxon test is calculated with all compounds in the cluster based on the weighting procedure described above. We used a strict threshold of p-value < 0.0001 to define a statistically significant difference between allosteric and competitive distributions; this was to reduce the likelihood that the differences were a coincidental artifact of having two large datasets and many comparisons of physical properties. These tests were implemented through R-Statistics (version 3.2.2) using the Wilcoxon Test in the R stats package [51]. Functions *svydesign* and *svyranktest* in R’s survey package were used for the weighted Wilcoxon test.

#### Bootstrap sampling

Bootstrap sampling was performed to determine the errors and variability in the means and medians of the calculated physical properties. Here, 100,000 samples (on the order of the number of ligand clusters in each set) were generated by selecting from each bin with probabilities corrected for the size of the cluster. Straight random sampling was used for the analysis using cluster centers. The bin widths for the weighted histograms were again 1 for descriptors with discrete values and 0.001 for continuous variables. The distributions of the mean and median from each sample were computed, and the 95% confidence intervals (95%ci) were then defined as the range of 2.5% to 97.5% of those distributions. We required all statistically significant differences to have no overlap in the 95%ci of the medians (in addition to Wilcoxon p-values < 0.0001 for the analysis above). It should be noted that all trends found to be significant in our data met the criteria by both weighted analysis and by center-of-cluster analysis (of course, the exact values for the means and medians were slightly different based on which analysis was used).

### Displaying the chemical similarity between the allosteric and competitive datasets

ChemTreeMap [52] was used to view the chemical space of the structures and display the chemical similarities in the two sets. ChemTreeMap organizes molecules in a hierarchical tree structure to convey molecular similarity information by combining extended connectivity fingerprint and a neighbor-joining algorithm. With hierarchical organization and color coding, ChemTreeMap is able to highlight the regions where chemical space is unique to each group of compounds. ECFP6 is used to characterize the molecules, and the hierarchical structure is determined from the T_c_ between molecules.

## Supporting information

S1 Allo-Comp-SMILES A listing of the smiles strings for all molecules used in this study. (XLSX)

S2 Allo-Comp-ChEMBL_assays A listing of all the assays from ChEMBL used to compile our dataset. (XLSX)

S1 Table Medians (95%ci) of the 29 physicochemical properties. Numbers in bold denote differences between allosteric and competitive compounds with p<0.0001 and no overlap in 95%ci of medians. **This analysis is done with single-level ligand clusters (i.e. no protein clustering).**
(DOCX)

S2 Table The 759 protein families for the allosteric set and 599 protein families for the competitive set (clustered at 60% sequence identity). The number of protein-ligand clusters is based on T_c_ = 0.6 clustering. Note: “None” is used when ASD or ChEMBL gave a FASTA sequence without a protein name.
(DOCX)

S3 Table Medians (95%ci) of the 29 physicochemical properties. Numbers in bold denote differences between allosteric and competitive compounds with p<0.0001 and no overlap in 95%ci of medians. **This analysis is done with the centers of the protein-ligand clusters.**
(DOCX)
