# Supplementary material for: Are there physicochemical differences between allosteric and competitive ligands?
Source: PLoS Comput Biol. 2017 Nov 10;13(11):e1005813. doi: 10.1371/journal.pcbi.1005813 (PMC5699844; doi:10.1371/journal.pcbi.1005813)
Supplement: S1 Table — Numbers in bold denote differences between allosteric and competitive compounds with p<0.0001 and no overlap in 95%ci of medians. This analysis is done with single-level ligand clusters (i.e. no protein clustering). (DOCX) [file pcbi.1005813.s003.docx]

**Table S1.** Medians (95%ci) of the 29 physicochemical properties. Numbers in bold denote differences between allosteric and competitive compounds with p<0.0001 and no overlap in 95%ci of medians. **This analysis is done with single-level ligand clusters (i.e. no protein clustering).**

|  | **60%/0.6** | | **75%/0.75** | | **90%/0.9** | | **100%/1.0** | |
| --- | --- | --- | --- | --- | --- | --- | --- | --- |
| **Properties** | Allosteric | Competitive | Allosteric | Competitive | Allosteric | Competitive | Allosteric | Competitive |
| a_heavy | 25±(0) | 25±(0) | 27±(0) | 26±(1) | 29±(0) | 28±(0) | 29±(0) | 29±(1) |
| a_aro | 12±(0) | 12±(0) | 12±(0) | 12±(0) | **15±(0)** | **12±(0)** | **15±(1)** | **12±(0)** |
| **a_aro/HA** | **0.5±(0)** | **0.46±(0.01)** | **0.5±(0)** | **0.458±(0.008)** | **0.5±(0)** | **0.444±(0.006)** | **0.50±(0.01)** | **0.43±(0.01)** |
| a_acc | **3±(0)** | **4±(0)** | 4±(1) | 4±(0) | 4±(0) | 4±(0) | 4±(0) | 4±(0) |
| **a_acc/HA** | **0.133±(0)** | **0.147±(0.004)** | **0.133±(0.001)** | **0.143±(0.003)** | **0.1364±(0.0008)** | **0.143±(0.004)** | **0.1373±(0.0009)** | **0.147±(0.004)** |
| **a_don** | **1±(0)** | **2±(0)** | **1±(0)** | **2±(0)** | **1±(0)** | **2±(0)** | **1±(0)** | **2±(0)** |
| **a_don/HA** | **0.042±(0.002)** | **0.080±(0.003)** | **0.04±(0)** | **0.074±(0.003)** | **0.04±(0)** | **0.074±(0.003)** | **0.036±(0)** | **0.077±(0.003)** |
| a_acid | 0±(0) | 0±(0) | 0±(0) | 0±(0) | 0±(0) | 0±(0) | 0±(0) | 0±(0) |
| a_acid/HA | 0±(0) | 0±(0) | 0±(0) | 0±(0) | 0±(0) | 0±(0) | 0±(0) | 0±(0) |
| a_base | 0±(0) | 0±(0) | 0±(0) | 0±(0) | 0±(0) | 0±(0) | 0±(0) | 0±(0) |
| a_base/HA | 0±(0) | 0±(0) | 0±(0) | 0±(0) | 0±(0) | 0±(0) | 0±(0) | 0±(0) |
| **b_count** | **45±(1)** | **47±(1)** | **48±(0)** | **50±(1)** | **52±(0)** | **54±(1)** | **53±(0)** | **55±(1)** |
| **b_count/HA** | **1.821±(0.005)** | **1.90±(0.01)** | **1.81±(0.003)** | **1.917±(0.008)** | **1.828±(0.001)** | **1.927±(0.007)** | **1.839±(0.005)** | **1.935±(0.006)** |
| b_ar | 12±(0) | 12±(0) | 12±(0) | 12±(0) | **16±(0)** | **12±(0)** | **16±(0)** | **12±(0)** |
| **b_ar/HA** | **0.5±(0)** | **0.46±(0.02)** | **0.515±(0.003)** | **0.46±(0.01)** | **0.5±(0)** | **0.450±(0.008)** | **0.5±(0)** | **0.44±(0.02)** |
| b_1rotN | 4±(0) | 5±(1) | **4±(0)** | **5±(0)** | 5±(0) | 5±(1) | **5±(0)** | **6±(0)** |
| **b_1rotN/HA** | **0.167±(0)** | **0.182±(0.003)** | **0.167±(0)** | **0.185±(0.003)** | **0.172±(0.001)** | **0.192±(0.002)** | **0.176±(0.002)** | **0.200±(0.006)** |
| FCharge | 0±(0) | 0±(0) | 0±(0) | 0±(0) | 0±(0) | 0±(0) | 0±(0) | 0±(0) |
| FCharge/HA | 0±(0) | 0±(0) | 0±(0) | 0±(0) | 0±(0) | 0±(0) | 0±(0) | 0±(0) |
| **SlogP** | **3.54±(0.03)** | **3.26±(0.09)** | **3.70±(0.02)** | **3.42±(0.07)** | **3.89±(0.01)** | **3.56±(0.05)** | **3.95±(0.01)** | **3.52±(0.05)** |
| **a_nC/HA** | **0.733±(0.002)** | **0.741±(0.004)** | **0.735±(0.002)** | **0.750±(0.007)** | **0.733±(0.002)** | **0.744±(0.006)** | **0.731±(0.003)** | **0.742±(0.002)** |
| logS | -4.61±(0.04) | -4.6±(0.1) | -4.84±(0.02) | -4.80±(0.07) | **-5.16±(0.02)** | **-5.09±(0.05)** | -5.29±(0.02) | -5.16±(0.05) |
| chiral | 0±(0) | 0±(0) | 0±(0) | 1±(1) | **0±(0)** | **1±(0)** | **0±(0)** | **1±(0)** |
| chiral/HA | 0±(0) | 0±(0) | 0±(0) | 0.02±(0.02) | **0±(0)** | **0.030±(0.002)** | **0±(0)** | **0.032±(0.001)** |
| rings | 3±(0) | 3±(0) | 3±(0) | 3±(0) | **4±(0)** | **3±(0)** | **4±(0)** | **3±(0)** |
| lip_druglike | 1±(0) | 1±(0) | 1±(0) | 1±(0) | 1±(0) | 1±(0) | 1±(0) | 1±(0) |
| lip_violation | 0±(0) | 0±(0) | 0±(0) | 0±(0) | 0±(0) | 0±(0) | 0±(0) | 0±(0) |
| opr_leadlike | 1±(0) | 1±(0) | 1±(0) | 1±(0) | 1±(0) | 1±(0) | 1±(0) | 1±(0) |
| opr_violation | 0±(0) | 1±(1) | **0±(0)** | **1±(0)** | 1±(0) | 1±(0) | 1±(0) | 1±(0) |
