# Supplementary material for: Are there physicochemical differences between allosteric and competitive ligands?
Source: PLoS Comput Biol. 2017 Nov 10;13(11):e1005813. doi: 10.1371/journal.pcbi.1005813 (PMC5699844; doi:10.1371/journal.pcbi.1005813)
Supplement: S2 Table — The number of protein-ligand clusters is based on Tc = 0.6 clustering. Note: “None” is used when ASD or ChEMBL gave a FASTA sequence without a protein name. (DOCX) [file pcbi.1005813.s004.docx]

**Table S2.** The 759 Protein Families for the Allosteric Set and 599 Protein Families for the Competitive Set (clustered at 60% sequence identity). The number of protein-ligand clusters is based on T_c_ = 0.6 clustering. Note: “None” is used when ASD or ChEMBL gave a FASTA sequence without a protein name.

| **Allosteric Targets** | **Ligands** | **Pro-Lig Clusters** | **Competitive Targets** | **Ligands** | **Pro-Lig Clusters** |
| --- | --- | --- | --- | --- | --- |
| Metabotropic glutamate receptor 5 | 14837 | 1949 | Estrogen receptor alpha | 636 | 184 |
| Pyruvate kinase isozyme R/L | 9967 | 271 | Dopamine D2 receptor | 499 | 170 |
| mGluR3 | 8666 | 1200 | Kappa opioid receptor | 390 | 53 |
| Cytochrome P450 NF-25 | 5382 | 54 | Acyl-CoA synthase | 369 | 309 |
| Alpha-7 nicotinic receptor | 4990 | 720 | Endothelin receptor ET-A | 361 | 44 |
| Muscarinic acetylcholine receptor M1 | 4976 | 968 | HERG | 263 | 69 |
| Non-enzyme | 3884 | 385 | Histamine H3 receptor | 243 | 74 |
| Gamma-aminobutyric acid receptor subunit gamma-2 | 3494 | 355 | Thromboxane A2 receptor | 227 | 52 |
| GABA-A receptor beta-3 subunit | 3467 | 345 | Menin/Histone-lysine N-methyltransferase MLL | 223 | 202 |
| Cannabinoid CB1 receptor | 3253 | 249 | Thrombin | 221 | 82 |
| NMD-R1 | 3233 | 401 | Aldo-keto-reductase family 1 member C3 | 215 | 37 |
| MAPK/ERK kinase 1 | 2872 | 290 | Androgen Receptor | 212 | 75 |
| mGluR8 | 2823 | 502 | Monoamine oxidase A | 205 | 75 |
| PKA C-beta | 2402 | 116 | Adenosine A1 receptor | 192 | 46 |
| GABA receptor alpha-2 subunit | 2349 | 351 | Progesterone receptor | 191 | 63 |
| FBPase 1 | 2237 | 135 | Inhibitor of apoptosis protein 3 | 181 | 54 |
| AMPK subunit alpha-2 | 2166 | 232 | Dopamine D4 receptor | 170 | 54 |
| Adenosine A3 receptor | 1970 | 302 | Dopamine transporter | 164 | 55 |
| Proto-oncogene c-Akt | 1932 | 136 | Retinoid X receptor gamma | 160 | 23 |
| ABPP | 1880 | 355 | Peroxisome proliferator-activated receptor alpha | 153 | 29 |
| GluA3 | 1873 | 447 | Integrin alpha-IIb/beta-3 | 153 | 38 |
| Brain cyclic nucleotide-gated channel 2 | 1619 | 198 | MAP kinase p38 alpha | 151 | 38 |
| L-glutamine amidohydrolase | 1560 | 166 | 11-beta-hydroxysteroid dehydrogenase 1 | 150 | 30 |
| P80 | 1453 | 173 | Alpha-1b adrenergic receptor | 139 | 46 |
| AMPK subunit beta-1 | 1444 | 228 | Glucocorticoid receptor | 133 | 38 |
| CAMP-dependent protein kinase type II-beta regulatory chain | 1442 | 115 | Serine/threonine-protein kinase AKT | 124 | 17 |
| Tissue-specific extinguisher 1 | 1442 | 115 | Renin | 122 | 19 |
| Sodium channel, voltage-gated, type I, alpha polypeptide | 1422 | 149 | Cholecystokinin B receptor | 113 | 17 |
| PK-1 | 1245 | 275 | FK506 binding protein 4 | 111 | 19 |
| GABABR1 | 1222 | 347 | Translocator protein | 107 | 35 |
| GABA-B-R2 | 1221 | 345 | Melanocortin receptor (M4 and M5) | 100 | 14 |
| Non-enzyme | 1166 | 322 | Mineralocorticoid receptor | 94 | 49 |
| RNA-directed RNA polymerase | 1159 | 275 | FK506-binding protein 1A | 93 | 9 |
| Cell division protein kinase 2 | 1078 | 217 | Gonadotropin-releasing hormone receptor | 91 | 13 |
| None | 1043 | 157 | Melatonin receptor | 82 | 30 |
| Follicle stimulating hormone receptor | 1037 | 113 | Serine/threonine-protein kinase Aurora-C | 79 | 8 |
| PtdIns-3-kinase subunit p110-gamma | 1008 | 268 | Signal transducer and activator of transcription 3 | 78 | 3 |
| Nicotinic acetylcholine receptor alpha4/beta2/alpha5 | 932 | 326 | G protein-coupled receptor 44 | 76 | 11 |
| CAMP-specific phosphodiesterase PDE4D6 | 899 | 201 | Melanin-concentrating hormone receptor 1 | 75 | 17 |
| D-fructose-1,6-bisphosphate 1-phosphohydrolase | 894 | 136 | Acetylcholinesterase | 75 | 56 |
| Hyperpolarization-activated (Ih) channel | 809 | 198 | Serine/threonine-protein kinase Chk1 | 72 | 33 |
| Neuronal nicotinic acetylcholine receptor alpha-4 subunit | 800 | 302 | Cyclooxygenase-2 | 70 | 16 |
| Avian erythroblastic leukemia viral (v-erb-b) oncogene homolog | 762 | 239 | Cytochrome P450 19A1 | 69 | 41 |
| Drosophila relative of ERBB | 755 | 232 | Cannabinoid receptor | 68 | 16 |
| Glutamate [NMDA] receptor subunit epsilon 1 | 728 | 118 | Baculoviral IAP repeat-containing protein 3 | 68 | 8 |
| Protein kinase AMP-activated gamma | 722 | 228 | Muscarinic acetylcholine receptor M1 | 68 | 44 |
| None | 722 | 228 | Prostanoid EP2 receptor | 67 | 25 |
| Presenilin-2 NTF subunit | 627 | 283 | PI3-kinase p110-beta subunit | 67 | 31 |
| RT | 590 | 219 | Retinoic acid receptor alpha | 66 | 10 |
| MMP-13 | 575 | 124 | Neurokinin 2 receptor | 66 | 17 |
| DNA polymerase beta | 549 | 107 | Serine/threonine-protein kinase Aurora-A | 64 | 8 |
| PN3 | 527 | 175 | Glandular kallikrein | 59 | 38 |
| Non-enzyme | 471 | 61 | Beta-glucocerebrosidase | 59 | 36 |
| Hsp90 | 458 | 99 | Carbonic anhydrase II | 59 | 41 |
| FBPase class 1 | 447 | 136 | Cannabinoid CB1 receptor | 58 | 15 |
| G-protein coupled receptor 71 | 438 | 229 | Angiotensin II receptor | 56 | 10 |
| Sweet taste receptor T1R3 | 438 | 229 | Beta-2 adrenergic receptor | 56 | 21 |
| S1P receptor Edg-3 | 419 | 124 | Apoptosis regulator Bcl-X | 56 | 17 |
| EK4 | 414 | 82 | Dihydrofolate reductase | 56 | 13 |
| HPDK1 | 405 | 162 | Apoptosis regulator Bcl-2 | 55 | 12 |
| Heat shock 70 kDa protein | 404 | 55 | Dopamine D1 receptor | 55 | 25 |
| p21-activated kinase 1 | 397 | 157 | Histamine H4 receptor | 53 | 27 |
| GLP-1 receptor | 395 | 202 | GABA-A receptor; anion channel | 53 | 13 |
| GL-R | 394 | 178 | Glutamate (NMDA) receptor subunit zeta 1 | 53 | 42 |
| Integrin beta-1 | 394 | 38 | Casein kinase II alpha | 52 | 10 |
| GR | 388 | 54 | Rap guanine nucleotide exchange factor 4 | 52 | 27 |
| Kinesin-like spindle protein HKSP | 388 | 181 | Serotonin 1a (5-HT1a) receptor | 50 | 19 |
| Prealbumin | 379 | 115 | Acrosin | 50 | 18 |
| 5-hydroxytryptamine receptor 3 | 357 | 74 | Histone deacetylase 3/Nuclear receptor corepressor 2 (HDAC3/NCoR2) | 49 | 9 |
| None | 351 | 46 | Histamine H2 receptor | 48 | 33 |
| Biotin carboxylase | 340 | 117 | Penicillin-binding protein 2 | 48 | 3 |
| Rapamycin and FKBP12 target 1 | 297 | 86 | Ras-related C3 botulinum toxin substrate 1 | 47 | 16 |
| Non-structural protein 5 | 289 | 136 | Hepatocyte growth factor receptor | 47 | 20 |
| Bcr/c-abl oncogene protein | 284 | 37 | Ryanodine receptor 1 | 47 | 17 |
| Parathyroid cell calcium-sensing receptor | 281 | 51 | Luciferin 4-monooxygenase | 44 | 10 |
| Beta globin chain | 278 | 50 | Angiotensin II type 2 (AT-2) receptor | 42 | 3 |
| Hemoglobin alpha chain | 278 | 50 | Histone deacetylase 9 | 42 | 7 |
| Ribonucleotide reductase R1 subunit 1 | 271 | 49 | Induced myeloid leukemia cell differentiation protein Mcl-1 | 42 | 9 |
| GRO/MGSA receptor | 265 | 100 | Receptor-type tyrosine-protein phosphatase beta | 42 | 41 |
| Serotonin receptor 2A | 264 | 92 | Serotonin 7 (5-HT7) receptor | 41 | 14 |
| Tyrosine kinase non-receptor protein 2 | 262 | 57 | Opioid receptor | 40 | 12 |
| P2X4 | 258 | 48 | PI3-kinase p110-alpha subunit | 39 | 19 |
| ATP receptor | 249 | 26 | p53-binding protein Mdm-2 | 38 | 6 |
| Sigma1-receptor (sterol-isomerase like protein) | 243 | 29 | Trypanothione reductase | 38 | 21 |
| Apoptosis inhibitor survivin | 235 | 35 | Matrix metalloproteinase 13 | 38 | 14 |
| CD49 antigen-like family member E | 234 | 26 | Cyclophilin A | 38 | 7 |
| Protein-tyrosine phosphatase 1B | 215 | 24 | Tumour suppressor p53/oncoprotein Mdm2 | 37 | 2 |
| MC5-R | 214 | 22 | Anandamide amidohydrolase | 37 | 12 |
| 5-HT1A | 211 | 54 | Cytochrome P450 2D6 | 37 | 13 |
| Bruton tyrosine kinase | 211 | 96 | Serotonin 2c (5-HT2c) receptor | 37 | 20 |
| Serotonin receptor 1A | 210 | 53 | SHC-transforming protein 1 | 36 | 3 |
| None | 200 | 40 | Platelet activating factor receptor | 36 | 17 |
| None | 187 | 36 | Vitamin D receptor | 36 | 14 |
| IDH1 | 182 | 17 | Monoglyceride lipase | 36 | 12 |
| Phosphatidylinositol 3-kinase p100 subunit | 182 | 45 | Cathepsin L2 | 35 | 12 |
| B2R | 176 | 53 | Galectin-9 | 35 | 17 |
| Integrase | 175 | 92 | Ghrelin receptor | 35 | 5 |
| CD49b | 159 | 12 | Galectin-3 | 35 | 17 |
| cGSPDE | 158 | 33 | Polymerase acidic protein | 34 | 21 |
| MAP kinase 14 | 151 | 58 | Coagulation factor X | 34 | 10 |
| HIV-1 PR | 138 | 41 | T-cell protein-tyrosine phosphatase | 34 | 15 |
| Non-enzyme | 136 | 49 | Macrophage colony stimulating factor receptor | 34 | 7 |
| Adenosylcobalamin-dependent ribonucleoside-triphosphate reductase | 136 | 49 | Histamine H1 receptor | 33 | 26 |
| Ribonucleoside-diphosphate reductase 2 subunit alpha | 136 | 49 | Disks large homolog 4 | 33 | 10 |
| Ribonucleotide reductase, B12-dependent | 136 | 49 | C5a anaphylatoxin chemotactic receptor | 32 | 5 |
| Ribonucleoside-diphosphate reductase 1 subunit alpha | 135 | 49 | Adenosine A2a receptor | 32 | 20 |
| Ribonucleotide reductase small chain | 135 | 49 | Galectin-7 | 32 | 16 |
| Janus kinase 1 | 134 | 44 | Tyrosine-protein kinase receptor FLT3 | 32 | 9 |
| Glutamate [NMDA] receptor subunit epsilon 3 | 131 | 15 | Mu opioid receptor | 32 | 7 |
| Non-enzyme | 130 | 17 | Vascular endothelial growth factor receptor 1 | 32 | 4 |
| CPT1-M | 128 | 8 | Heat shock protein HSP 90-alpha | 31 | 13 |
| Serine/threonine-protein kinase aurora-A | 127 | 8 | Adenylate kinase 3 alpha like 1 | 31 | 10 |
| SMO | 126 | 26 | Phosphodiesterase 10A | 31 | 5 |
| None | 124 | 28 | Galectin-8 | 31 | 14 |
| Dopamine D3 receptor | 120 | 53 | IgG receptor FcRn large subunit p51 | 30 | 1 |
| nPKC-epsilon | 119 | 36 | Histone-lysine N-methyltransferase, H3 lysine-79 specific | 30 | 10 |
| SL3/AKV core-binding factor alpha B subunit | 119 | 24 | Beta-1 adrenergic receptor | 30 | 16 |
| GPR-CY6 | 113 | 40 | Aminopeptidase N | 30 | 18 |
| SCFR | 109 | 14 | Atrial natriuretic peptide receptor C | 27 | 1 |
| TER ATPase | 109 | 33 | 3-dehydroquinate dehydratase | 27 | 12 |
| Voltage-gated potassium channel subunit Kv11.1 | 109 | 14 | Adenylate kinase 2 | 27 | 7 |
| IKK-B | 107 | 14 | Eukaryotic translation initation factor | 26 | 5 |
| Porphobilinogen synthase | 105 | 25 | Serotonin 1d (5-HT1d) receptor | 26 | 4 |
| SDH | 97 | 63 | 3-phosphoinositide dependent protein kinase-1 | 26 | 22 |
| Calcium pump 1 | 96 | 13 | Peptide deformylase mitochondrial | 26 | 14 |
| RGS4 | 91 | 37 | Thymidine kinase, cytosolic | 26 | 14 |
| PGE2 receptor EP2 subtype | 89 | 20 | WD repeat-containing protein 5 | 25 | 5 |
| hSIRT1 | 88 | 69 | Tyrosine-protein kinase Lyn | 25 | 10 |
| NKR | 86 | 44 | Serine/threonine-protein kinase PIM1 | 25 | 11 |
| GLP-2 receptor | 85 | 28 | Pyruvate kinase isozymes R/L | 24 | 6 |
| UBC4/5 homolog | 85 | 31 | Prostanoid IP receptor | 24 | 5 |
| Free fatty acid activated receptor 2 | 84 | 38 | CpG DNA methylase | 24 | 17 |
| GSase | 84 | 22 | Serotonin (5-HT) receptor | 24 | 14 |
| GDH 1 | 83 | 21 | Neurotensin receptor 1 | 24 | 4 |
| PTH/PTHrP type I receptor | 83 | 4 | Serine/threonine-protein kinase Chk2 | 24 | 14 |
| Exoribonuclease H | 81 | 63 | Glucagon receptor | 24 | 5 |
| Putative uncharacterized protein TRT1 | 81 | 49 | HLA class II histocompatibility antigen DRB3-1 | 23 | 6 |
| Serotonin transporter | 81 | 40 | Calcitonin gene-related peptide 1 | 23 | 11 |
| TP2 | 81 | 49 | Kallikrein 5 | 23 | 12 |
| None | 80 | 34 | Peptidyl-glycine alpha-amidating monooxygenase | 23 | 18 |
| gp68 | 79 | 22 | Peptide deformylase | 23 | 11 |
| CD11 antigen-like family member A | 78 | 32 | Trypsin I | 23 | 7 |
| US28 | 77 | 50 | Neuronal acetylcholine receptor; alpha4/beta2 | 23 | 7 |
| G-protein coupled receptor HG11 | 76 | 3 | Prostanoid EP4 receptor | 22 | 6 |
| Flavin-containing amine oxidase domain-containing protein 2 | 75 | 24 | CD209 antigen | 22 | 10 |
| FPS | 74 | 54 | Sialidase | 21 | 14 |
| AChE | 71 | 23 | Mitogen-activated protein kinase kinase kinase 5 | 21 | 8 |
| Thrombin receptor | 68 | 26 | Fatty acid-binding protein, liver | 21 | 20 |
| Non-enzyme | 66 | 15 | Prostanoid EP3 receptor | 21 | 5 |
| GluK1 | 65 | 30 | Phosphodiesterase 4B | 21 | 9 |
| Nuclear receptor subfamily 3 group C member 4 | 65 | 32 | Autotaxin | 21 | 12 |
| Serine/threonine protein kinase | 64 | 9 | 3-dehydroquinate dehydratase | 20 | 8 |
| None | 64 | 28 | Somatostatin receptor | 20 | 2 |
| Burkitt lymphoma receptor 1 | 63 | 4 | Complement C1s | 20 | 19 |
| CRF-R1 | 63 | 8 | Thymidylate synthase | 20 | 7 |
| Short transient receptor potential channel 4 | 62 | 57 | Plasminogen | 20 | 20 |
| P94 | 60 | 17 | Transthyretin | 20 | 18 |
| Serpin C1 | 57 | 36 | Furin | 20 | 5 |
| Chloride channel Ka | 55 | 18 | Prostanoid EP1 receptor | 20 | 4 |
| GC-C | 54 | 12 | Monoacylglycerol lipase ABHD6 | 20 | 3 |
| JNK-46 | 54 | 37 | Matrix metalloproteinase 9 | 20 | 4 |
| Scatter factor | 52 | 15 | Glycogen phosphorylase, muscle form | 19 | 12 |
| Thrombin light chain | 51 | 30 | Vanilloid receptor | 19 | 5 |
| Neurotrophic tyrosine kinase receptor type 1 | 50 | 33 | Beta amyloid A4 protein | 19 | 5 |
| TP | 50 | 9 | S-adenosylmethionine synthetase gamma form | 19 | 9 |
| Voltage-gated calcium channel subunit alpha Cav3.2 | 50 | 23 | Gamma-amino-N-butyrate transaminase | 19 | 18 |
| AK | 49 | 9 | Thymidine kinase | 19 | 10 |
| TGase-2 | 49 | 18 | G-protein coupled bile acid receptor 1 | 19 | 6 |
| Glutamate [NMDA] receptor subunit epsilon 4 | 48 | 5 | Growth factor receptor-bound protein 2 | 19 | 8 |
| Helicase with RNase motif | 40 | 17 | Tyrosine-protein kinase TYK2 | 18 | 6 |
| None | 40 | 8 | Muscarinic acetylcholine receptor M5 | 18 | 8 |
| Alpha-glucosidase I | 39 | 10 | Adhesin protein fimH | 18 | 5 |
| Plasmin | 38 | 21 | c-Jun N-terminal kinase 2 | 18 | 4 |
| ATP receptor | 37 | 23 | CD22 | 18 | 2 |
| 1,25-dihydroxyvitamin D3 receptor | 36 | 4 | Lipoxygenase | 17 | 13 |
| 5-HT4 | 36 | 8 | Kallikrein 7 | 17 | 7 |
| Beta-Ketoacyl-acyl carrier protein reductase | 36 | 17 | Alpha-chymotrypsin | 17 | 11 |
| DNA-binding factor KBF1 | 36 | 18 | Botulinum neurotoxin type A | 17 | 11 |
| Glycine receptor 48 kDa subunit | 36 | 14 | Rho-associated protein kinase 1 | 17 | 17 |
| Glycine receptor 58 kDa subunit | 36 | 14 | Tyrosinase | 17 | 11 |
| Nuclear factor of kappa light polypeptide gene enhancer in B-cells 3 | 36 | 18 | DNA topoisomerase I | 16 | 3 |
| Non-enzyme | 35 | 24 | Vitamin K-dependent protein C | 16 | 5 |
| ALADH | 35 | 24 | Integrin alpha-V/beta-5 | 16 | 8 |
| ALADH | 35 | 24 | Ribonuclease pancreatic | 16 | 7 |
| Porphobilinogen synthase | 35 | 24 | C-C motif chemokine 2 | 16 | 2 |
| Cell surface glycoprotein MAC-1 subunit alpha | 34 | 33 | Leukocyte elastase | 16 | 10 |
| LIMK-2 | 34 | 12 | Serotonin 6 (5-HT6) receptor | 16 | 7 |
| SAMDC | 34 | 18 | Choline acetylase | 16 | 15 |
| SAMDC | 34 | 18 | Thymidylate kinase | 16 | 8 |
| Non-enzyme | 33 | 15 | Cathepsin B | 15 | 12 |
| CHK1 | 33 | 7 | Vascular endothelial growth factor receptor 2 | 15 | 7 |
| MurI | 33 | 15 | Histone deacetylase 6 | 15 | 8 |
| Epoxide hydratase | 31 | 28 | Platelet-derived growth factor receptor beta | 15 | 5 |
| ET-A | 30 | 28 | Histone deacetylase 10 | 15 | 8 |
| Rd | 30 | 15 | Serine/threonine-protein kinase PLK1 | 15 | 5 |
| Tumor necrosis factor-alpha receptor | 30 | 16 | Activin receptor type-1B | 15 | 3 |
| IP-10 receptor | 29 | 9 | Histone deacetylase 11 | 14 | 7 |
| Kappa opioid receptor | 29 | 21 | Fibroblast activation protein alpha | 14 | 11 |
| NADP-malic enzyme 2 | 29 | 14 | Fibroblast growth factor receptor 1 | 14 | 2 |
| GABA-A receptor; anion channel | 28 | 6 | Insulin-like growth factor I receptor | 14 | 6 |
| Arachidonate omega-6 lipoxygenase | 27 | 8 | Histone deacetylase 8 | 14 | 8 |
| c-H-ras | 27 | 19 | Breast cancer type 1 susceptibility protein | 14 | 2 |
| GCS-alpha-2 | 27 | 12 | Nuclear receptor ROR-alpha | 14 | 11 |
| GCS-beta-3 | 27 | 12 | Asialoglycoprotein receptor 1 | 14 | 9 |
| Phosphohexokinase | 27 | 8 | Serotonin 2b (5-HT2b) receptor | 14 | 7 |
| Aspartate transcarbamylase | 26 | 10 | Beta-lactamase | 14 | 10 |
| IDH | 26 | 17 | Adenylate kinase 1 | 13 | 6 |
| Isocitrate dehydrogenase [NAD] subunit alpha, mitochondrial | 26 | 17 | Leukotriene B4 receptor 1 | 13 | 7 |
| Isocitric dehydrogenase subunit gamma | 26 | 17 | Prostanoid FP receptor | 13 | 2 |
| NAD(+)-specific ICDH subunit beta | 26 | 17 | Apoptosis regulator Bcl-W | 13 | 3 |
| Nicotinic acid receptor | 26 | 6 | Adrenergic receptor alpha-2 | 13 | 7 |
| AtMEPCT | 25 | 16 | Receptor protein-tyrosine kinase erbB-2 | 13 | 4 |
| nPKC-zeta | 25 | 5 | Cytochrome P450 2C9 | 12 | 8 |
| P60-Src | 25 | 17 | P-selectin | 12 | 7 |
| PLD | 25 | 10 | Subtilisin | 12 | 12 |
| UDP/CysLT receptor | 25 | 24 | Dihydroorotate dehydrogenase | 12 | 3 |
| UK | 25 | 3 | Tubulin | 12 | 6 |
| Mitogen-activated protein kinase phosphatase 3 | 24 | 6 | Dual-specificity tyrosine-phosphorylation regulated kinase 1A | 12 | 7 |
| P2U purinoceptor 1 | 24 | 9 | Inositol 1,4,5-trisphosphate receptor type 3 | 11 | 8 |
| Cannabinoid CB2 receptor | 23 | 4 | Cytochrome P450 3A4 | 11 | 8 |
| Endoribonuclease | 23 | 8 | Alpha-L-fucosidase I | 11 | 10 |
| ICE-like apoptotic protease 6 | 23 | 5 | Subtilisin/kexin type 6 | 11 | 4 |
| Threonine aspartase subunit alpha | 23 | 12 | Thymidylate synthase | 11 | 2 |
| 6-phosphofructokinase, platelet type | 22 | 10 | Low molecular weight phosphotyrosine protein phosphatase | 11 | 3 |
| Prostanoid FP receptor | 22 | 3 | Purine nucleoside phosphorylase | 11 | 10 |
| CBF-beta | 21 | 16 | Beta-secretase 1 | 11 | 5 |
| CUL-5 | 21 | 12 | Ornithine decarboxylase | 11 | 6 |
| Alpha-actin-1 | 20 | 8 | Xanthine dehydrogenase | 11 | 10 |
| OTCase | 20 | 9 | Histone deacetylase 7 | 11 | 6 |
| PDC1 | 20 | 10 | Subtilisin | 11 | 11 |
| Sensory neuron-specific G-protein coupled receptor 1 | 20 | 6 | Sn1-specific diacylglycerol lipase beta | 10 | 3 |
| DNA deoxyribophosphodiesterase | 19 | 11 | Neurotrophic tyrosine kinase receptor type 2 | 10 | 4 |
| Exonuclease I | 19 | 11 | Glycogen synthase kinase-3 alpha | 10 | 8 |
| Focal adhesion kinase 1 | 19 | 10 | Bradykinin B1 receptor | 10 | 2 |
| Integrin alpha-4 | 19 | 12 | Glutathione reductase | 10 | 8 |
| Myosin II heavy chain | 19 | 18 | Seed lipoxygenase-1 | 10 | 4 |
| UGT1*1 | 19 | 6 | Bradykinin B2 receptor | 10 | 3 |
| VEGF Receptor 2 | 19 | 8 | MecA | 10 | 9 |
| A2 A adenosine receptor | 18 | 14 | Muscarinic acetylcholine receptor M2 | 10 | 5 |
| E-NPP 2 | 18 | 15 | Cytosol aminopeptidase | 9 | 7 |
| Phosphatidylcholine 2-acylhydrolase 1B | 18 | 4 | Coagulation factor XI | 9 | 4 |
| Tryptophan synthase beta chain | 18 | 14 | Epithelial discoidin domain-containing receptor 1 | 9 | 4 |
| hsAC | 17 | 16 | Matrix metalloproteinase 14 | 9 | 4 |
| Insulin-like growth factor I receptor | 17 | 6 | Leukocyte adhesion molecule-1 | 9 | 6 |
| Leukotriene A(4) hydrolase | 17 | 17 | Dihydrofolate reductase | 9 | 1 |
| PEPCase 1 | 17 | 15 | Coagulation factor VII | 9 | 3 |
| Tryptophan synthase alpha chain | 17 | 13 | Adrenergic receptor alpha-1 | 9 | 6 |
| Cu-NIR | 16 | 15 | Stem cell growth factor receptor | 9 | 4 |
| Estradiol receptor | 16 | 14 | Phosphodiesterase 7A | 9 | 4 |
| GnRH-R | 16 | 8 | Tyrosine-protein kinase ABL | 8 | 5 |
| Hydroxylamine reductase | 16 | 15 | 3-dehydroquinate dehydratase | 8 | 2 |
| Phospho-2-keto-3-deoxyheptonate aldolase | 16 | 4 | Phenylalanine-4-hydroxylase | 8 | 8 |
| SA | 16 | 13 | Phosphodiesterase 5A | 8 | 2 |
| SPAAT | 16 | 13 | Vasopressin V2 receptor | 8 | 3 |
| Non-enzyme | 15 | 3 | Testis-specific androgen-binding protein | 8 | 8 |
| CGMP-binding cGMP-specific phosphodiesterase | 15 | 4 | Leukotriene A4 hydrolase | 8 | 6 |
| G protein-coupled receptor kinase GRK6 | 15 | 5 | Thermolysin | 8 | 5 |
| Gelatinase B | 15 | 14 | D-amino-acid oxidase | 8 | 8 |
| ODC | 15 | 11 | Nitric oxide synthase, inducible | 8 | 4 |
| PEPCase | 15 | 14 | Serotonin 5a (5-HT5a) receptor | 8 | 2 |
| Transformation-related protein 53 | 15 | 6 | Vesicular glutamate transporter 3 | 8 | 8 |
| None | 15 | 12 | Cruzipain | 8 | 7 |
| Citrate cleavage enzyme | 14 | 6 | Ephrin type-A receptor 8 | 8 | 5 |
| Estrogen synthase | 14 | 6 | Human immunodeficiency virus type 1 protease | 8 | 6 |
| Homoserine dehydrogenase | 14 | 6 | Casein kinase I epsilon | 8 | 4 |
| Huff | 14 | 12 | Glyoxalase I | 8 | 5 |
| Malate dehydrogenase | 14 | 14 | Catechol O-methyltransferase | 8 | 8 |
| Malate dehydrogenase, decarboxylating | 14 | 14 | Heat shock protein HSP 60 | 8 | 5 |
| Threonine dehydratase | 14 | 5 | Max-like protein X | 8 | 4 |
| Aspartate carbamoyltransferase regulatory chain | 13 | 11 | Phosphodiesterase 11A | 8 | 2 |
| Aspartate carbamoyltransferase regulatory chain | 13 | 11 | Dual specificity mitogen-activated protein kinase kinase 1 | 8 | 2 |
| Beta2-adrenoceptor | 13 | 2 | Phosphodiesterase 3A | 7 | 2 |
| Glyceraldehyde-3-phosphate dehydrogenase (NADP+) | 13 | 11 | Cystine/glutamate transporter | 7 | 6 |
| Glyceraldehyde-3-phosphate dehydrogenase (Phosphorylating) | 13 | 11 | Phosphodiesterase 8A | 7 | 2 |
| GPDH | 13 | 11 | Indoleamine 2,3-dioxygenase | 7 | 5 |
| Monoamine oxidase type B | 13 | 11 | Acidic alpha-glucosidase | 7 | 7 |
| TS | 13 | 7 | Cyclin A2 | 7 | 3 |
| Non-enzyme | 12 | 3 | Serine/threonine-protein kinase PAK 3 | 7 | 2 |
| Bacteriophage N4 adsorption protein C | 12 | 4 | Tyrosine-protein kinase receptor UFO | 7 | 2 |
| CXC-R4 | 12 | 2 | Matrix metalloproteinase 7 | 7 | 2 |
| Hemocyanin A chain | 12 | 6 | Vitamin D-binding protein | 7 | 3 |
| Hemocyanin A chain | 12 | 6 | Phosphodiesterase 9A | 7 | 2 |
| L-1 | 12 | 10 | Glyceraldehyde-3-phosphate dehydrogenase, glycosomal | 7 | 1 |
| Mevalonate kinase | 12 | 3 | MAP kinase-interacting serine/threonine-protein kinase MNK1 | 7 | 2 |
| Nuclear receptor subfamily 3 group C member 3 | 12 | 7 | L-lactate dehydrogenase A chain | 7 | 6 |
| PA | 12 | 5 | Mycophenolic acid acyl-glucuronide esterase, mitochondrial | 7 | 4 |
| Protein Yama | 12 | 11 | Proteasome component C5 | 7 | 2 |
| U-PAR | 12 | 3 | Phosphodiesterase 2A | 7 | 2 |
| None | 12 | 6 | Tubulin alpha chain | 7 | 3 |
| None | 12 | 6 | Pho80/Pho85/Pho81 complex | 7 | 2 |
| CaMK-II subunit gamma | 11 | 9 | Phosphodiesterase 1A | 7 | 2 |
| N(1),N(8)-bis(glutathionyl)spermidine reductase | 11 | 2 | Integrin alpha-4 | 7 | 1 |
| Nuclear receptor subfamily 2 group B member 1 | 11 | 11 | Galanin receptor 1 | 6 | 1 |
| CK II alpha | 10 | 3 | Selectin E | 6 | 3 |
| Cytosine aminohydrolase | 10 | 3 | Alpha-1d adrenergic receptor | 6 | 2 |
| HD1 | 10 | 3 | Serine/threonine-protein kinase B-raf | 6 | 4 |
| IMP dehydrogenase 1 | 10 | 5 | Phosphotyrosine-protein phosphatase PTPB | 6 | 5 |
| Na(+)/K(+) ATPase alpha-1 subunit | 10 | 7 | Gamma-glutamyltranspeptidase 1 | 6 | 6 |
| Na(+)/K(+) ATPase subunit gamma | 10 | 7 | Bcl-2-related protein A1 | 6 | 3 |
| PDC | 10 | 10 | Equilibrative nucleoside transporter 1 | 6 | 2 |
| Probable ribonuclease P/MRP protein subunit POP5 | 10 | 6 | ALK tyrosine kinase receptor | 6 | 4 |
| Ribose-phosphate pyrophosphokinase 1 | 10 | 4 | Neutral cholesterol ester hydrolase 1 | 6 | 1 |
| RNase MRP protein subunit | 10 | 6 | Sphingosine kinase 1 | 6 | 6 |
| RNase P protein subunit | 10 | 6 | Cystinyl aminopeptidase | 6 | 5 |
| RNase P protein subunit | 10 | 6 | Proteasome Macropain subunit MB1 | 6 | 3 |
| RNase P protein subunit | 10 | 6 | Dual specificty protein kinase CLK1 | 6 | 2 |
| RNase P protein subunit DRpp40 | 10 | 6 | Myelin-associated glycoprotein | 6 | 2 |
| Sodium/potassium-dependent ATPase subunit beta-1 | 10 | 7 | Mannosidase 2 alpha 1 | 6 | 3 |
| None | 10 | 2 | Platelet-activating factor acetylhydrolase | 6 | 1 |
| ATP-dependent helicase E1 | 9 | 7 | Galanin receptor 2 | 6 | 1 |
| Beta-1 metal-binding globulin | 9 | 9 | UDP-glucuronosyltransferase 2B7 | 6 | 3 |
| CaM | 9 | 3 | Carboxylesterase 1D | 6 | 1 |
| Cathepsin O | 9 | 9 | Tyrosine-protein kinase TEC | 6 | 3 |
| Cytochrome b-562 | 9 | 9 | Arachidonate 12-lipoxygenase | 6 | 5 |
| Cytosolic 5'-nucleotidase II | 9 | 5 | 1-deoxy-D-xylulose-5-phosphate synthase | 6 | 5 |
| Exchange protein directly activated by cAMP 1 | 9 | 5 | Pantothenate synthetase | 6 | 4 |
| Inducible NOS | 9 | 7 | DNA polymerase alpha subunit | 6 | 4 |
| MOP-5 | 9 | 7 | Aminopeptidase B | 6 | 4 |
| Oxytocin-neurophysin 1 | 9 | 6 | Folylpoly-gamma-glutamate synthetase | 5 | 3 |
| Penicillin binding protein 2 prime | 9 | 7 | Chymotrypsin C | 5 | 3 |
| SSAO | 9 | 9 | Casein kinase I gamma 2 | 5 | 3 |
| TIS10 protein | 9 | 4 | Tryptophan 2,3-dioxygenase | 5 | 5 |
| Non-enzyme | 8 | 4 | Dual specificity tyrosine-phosphorylation-regulated kinase 1B | 5 | 3 |
| Alpha-1C adrenergic receptor | 8 | 6 | Cytochrome P450 1A2 | 5 | 5 |
| ATP-sulfurylase | 8 | 4 | Glycerol kinase | 5 | 1 |
| ATP-sulfurylase | 8 | 4 | Adenosine deaminase | 5 | 4 |
| Beta-thionase | 8 | 4 | Serine/threonine-protein kinase RAF | 5 | 3 |
| cAMP-GEFII | 8 | 8 | Protein kinase C eta | 5 | 4 |
| cGMP-dependent protein kinase I | 8 | 4 | Bromodomain adjacent to zinc finger domain protein 2B | 5 | 1 |
| Deoxycytidylate aminohydrolase | 8 | 4 | MAP/microtubule affinity-regulating kinase 2 | 5 | 2 |
| Deoxycytidylate aminohydrolase | 8 | 4 | Nicotinamide phosphoribosyltransferase | 5 | 4 |
| DNA-directed RNA polymerase subunit alpha | 8 | 3 | MAP kinase-activated protein kinase 2 | 5 | 3 |
| Heterogeneous nuclear ribonucleoprotein methyltransferase-like protein 3 | 8 | 6 | Uridine phosphorylase 1 | 5 | 3 |
| ICE-LAP3 | 8 | 7 | Tyrosine-protein phosphatase yopH | 5 | 4 |
| Insulysin | 8 | 3 | Glutathione S-transferase A1 | 5 | 3 |
| Lactose Repressor | 8 | 6 | Neutral alpha-glucosidase AB | 5 | 5 |
| N-acetylglucosamine-1-phosphate uridyltransferase | 8 | 4 | 2-dehydro-3-deoxyphosphooctonate aldolase | 5 | 4 |
| PP-1A | 8 | 3 | Glutathione reductase | 4 | 2 |
| Putative deoxycytidylate deaminase | 8 | 4 | Serine/threonine-protein kinase DCLK1 | 4 | 2 |
| Recombinase A | 8 | 2 | Proteasome Macropain subunit | 4 | 1 |
| RNA polymerase subunit beta | 8 | 3 | Beta-hexosaminidase | 4 | 2 |
| RNAP subunit alpha | 8 | 3 | Tyrosine-protein kinase receptor RET | 4 | 2 |
| RNAP subunit beta | 8 | 3 | Glutamate receptor ionotropic, kainate | 4 | 4 |
| UDP-N-acetylglucosamine pyrophosphorylase, putative | 8 | 4 | Fe(3+)-Zn(2+) purple acid phosphatase | 4 | 1 |
| None | 8 | 7 | Beta-3 adrenergic receptor | 4 | 2 |
| 235aa long hypothetical biotin--[acetyl-CoA-carboxylase] ligase | 7 | 4 | Bone morphogenetic protein receptor type-2 | 4 | 2 |
| Biotin repressor | 7 | 4 | Prolyl endopeptidase | 4 | 4 |
| Biotin--acetyl-CoA-carboxylase ligase | 7 | 4 | Focal adhesion kinase 1 | 4 | 2 |
| CAP | 7 | 4 | Dual specificity phosphatase Cdc25B | 4 | 4 |
| Caspase-6 subunit p11 | 7 | 7 | Inosine-5'-monophosphate dehydrogenase 2 | 4 | 1 |
| DegS | 7 | 2 | PI3-kinase p110-gamma subunit | 4 | 2 |
| GC-A | 7 | 7 | Beta-hexosaminidase subunit beta | 4 | 2 |
| G-protein coupled receptor 40 | 7 | 7 | DNA-dependent protein kinase | 4 | 4 |
| Solute carrier family 5 member 7 | 7 | 5 | Plasmepsin 2 | 4 | 1 |
| Stuart factor | 7 | 4 | Tyrosine-protein kinase TIE-2 | 4 | 2 |
| Tyrosine 3-hydroxylase | 7 | 4 | Diamine oxidase | 4 | 4 |
| Non-enzyme | 6 | 5 | Tyrosine-protein kinase FER | 4 | 1 |
| AE 1 | 6 | 5 | RocR | 4 | 2 |
| AMP deaminase isoform M | 6 | 6 | Inosine-5'-monophosphate dehydrogenase, probable | 4 | 3 |
| Coagulation factor XI | 6 | 6 | Ribosomal protein S6 kinase alpha 1 | 4 | 2 |
| Dihydropteroate synthase | 6 | 6 | Glutamate receptor ionotropic, AMPA 2 | 4 | 4 |
| Gamma-tubulin complex component 1 | 6 | 6 | Adenosylhomocysteinase | 4 | 3 |
| GNAT | 6 | 3 | Serine/threonine-protein kinase PLK4 | 4 | 2 |
| HTPA synthase | 6 | 2 | CaM-kinase kinase beta | 4 | 1 |
| Hydroxymethylbilane synthase | 6 | 2 | Kynureninase | 4 | 4 |
| p75 | 6 | 5 | Quinone reductase 2 | 4 | 3 |
| Phosphatidylinositol-specific phospholipase C | 6 | 2 | Lysophosphatidic acid receptor Edg-2 | 4 | 1 |
| Polyprotein | 6 | 2 | Uracil nucleotide/cysteinyl leukotriene receptor | 4 | 4 |
| PP2C-alpha | 6 | 2 | Vasoactive intestinal peptide receptor | 4 | 2 |
| Pyruvate dehydrogenase, lipoamide, kinase isozyme 2, mitochondrial | 6 | 5 | Kynurenine 3-monooxygenase | 4 | 4 |
| Sodium-dependent glutamate/aspartate transporter 1 | 6 | 3 | Phosphatidylinositol-4-phosphate 3-kinase C2 domain-containing beta polypeptide | 3 | 1 |
| Type II Citrate Synthases | 6 | 4 | S-ribosylhomocysteine lyase | 3 | 2 |
| 1.3.1.20</ecNumber | 5 | 4 | Mitogen-activated protein kinase kinase kinase 9 | 3 | 1 |
| Non-enzyme | 5 | 4 | Glyoxalase II | 3 | 2 |
| Alpha-2 adrenergic receptor subtype C2 | 5 | 4 | Asparagine synthetase | 3 | 3 |
| Beta-Carbonic Anhydrase | 5 | 5 | Tyrosine-protein kinase ZAP-70 | 3 | 1 |
| CK II beta | 5 | 3 | Carbepenem-hydrolyzing beta-lactamase KPC | 3 | 3 |
| DAHP synthase | 5 | 4 | Protein kinase C theta | 3 | 2 |
| D-alanyl-D-alanine carboxypeptidase | 5 | 3 | Serine/threonine-protein kinase 32C | 3 | 1 |
| GABA(C) receptor | 5 | 5 | Serine/threonine-protein kinase GAK | 3 | 2 |
| GLIC | 5 | 5 | Cell division cycle 2-like protein kinase 6 | 3 | 1 |
| Heat shock protein HslV | 5 | 4 | Vasopressin V1a receptor | 3 | 2 |
| ICE | 5 | 3 | Enoyl-[acyl-carrier-protein] reductase [NADH] | 3 | 3 |
| KDC | 5 | 4 | Succinyl-diaminopimelate desuccinylase | 3 | 3 |
| MIF | 5 | 3 | Alpha-glucosidase | 3 | 2 |
| PAS domain-containing protein 2 | 5 | 4 | Sucrase-isomaltase | 3 | 3 |
| PGDH | 5 | 5 | Hexokinase | 3 | 3 |
| Phosphoglycerate dehydrogenase | 5 | 5 | Chaperone activity of bc1 complex-like, mitochondrial | 3 | 1 |
| PLC-delta-1 | 5 | 1 | Protein-arginine N-methyltransferase 1 | 3 | 3 |
| PPAR-gamma | 5 | 3 | Mitogen-activated protein kinase kinase kinase 4 | 3 | 1 |
| PPDC | 5 | 4 | Isoprenylcysteine carboxyl methyltransferase | 3 | 2 |
| Pur regulon repressor | 5 | 5 | Protein kinase C alpha | 3 | 1 |
| Regulatory protein SIR2 homolog 3 | 5 | 4 | Probable low molecular weight protein-tyrosine-phosphatase | 3 | 3 |
| SK 1 | 5 | 3 | Serine/threonine-protein kinase 11 | 3 | 1 |
| TEM-5 | 5 | 5 | Bifunctional protein glmU | 3 | 3 |
| Trypanothione synthetase, putative | 5 | 4 | Phosphatidylinositol-5-phosphate 4-kinase type-2 beta | 3 | 1 |
| None | 5 | 2 | Penicillin-binding protein 2x | 3 | 2 |
| None | 5 | 2 | Serine/threonine-protein kinase PLK3 | 3 | 1 |
| None | 5 | 2 | Macrophage migration inhibitory factor | 3 | 3 |
| 6-DEB hydroxylase | 4 | 4 | NUAK family SNF1-like kinase 2 | 3 | 1 |
| Non-enzyme | 4 | 4 | DNA topoisomerase II alpha | 3 | 3 |
| Non-enzyme | 4 | 4 | Cyclin-dependent kinase 7 | 3 | 1 |
| Non-enzyme | 4 | 2 | Serine/threonine-protein kinase PAK 4 | 3 | 1 |
| Non-enzyme | 4 | 4 | Testis-specific serine/threonine-protein kinase 1 | 3 | 1 |
| Acute-phase response factor | 4 | 4 | Prostaglandin E synthase | 3 | 3 |
| ADP-glucose synthase | 4 | 4 | Phospho-2-dehydro-3-deoxyheptonate aldolase | 3 | 3 |
| AGPase B | 4 | 4 | Voltage-gated L-type calcium channel | 3 | 3 |
| Alpha-ketoglutarate decarboxylase | 4 | 2 | Cyclin-dependent kinase 9 | 3 | 1 |
| Arginine hydroxamate resistance protein | 4 | 2 | Heparanase | 3 | 1 |
| ATP-PRT | 4 | 4 | TRAIL receptor-1 | 3 | 3 |
| ATP-PRT | 4 | 4 | Sphingosine kinase 2 | 3 | 3 |
| ATP-PRTase | 4 | 4 | Penicillin-binding protein 2B | 3 | 2 |
| Branched-chain alpha-ketoacid dehydrogenase kinase | 4 | 2 | Acetylcholine receptor protein delta chain | 3 | 2 |
| CAP | 4 | 3 | Terminal deoxynucleotidyltransferase | 3 | 3 |
| Carbamoyl-phosphate synthetase ammonia chain | 4 | 4 | Penicillin-binding protein 1A | 3 | 2 |
| Carbamoyl-phosphate synthetase glutamine chain | 4 | 4 | Pyridoxal kinase | 3 | 3 |
| CASP-2 | 4 | 3 | Urease | 3 | 3 |
| CCK2-R | 4 | 3 | Trace amine-associated receptor 1 | 3 | 3 |
| Chymotrypsinogen B2 | 4 | 1 | P-selectin | 3 | 3 |
| CXXC-type zinc finger protein 9 | 4 | 4 | Serine/threonine-protein kinase PCTAIRE-1 | 3 | 1 |
| EK | 4 | 3 | Dual specificity protein kinase CLK2 | 3 | 2 |
| ER-beta | 4 | 4 | Neurotensin receptor | 3 | 2 |
| FADD-like ICE | 4 | 4 | Riboflavin-binding protein | 3 | 3 |
| GroEL | 4 | 2 | Bile salt export pump | 3 | 1 |
| Guanine nucleotide-binding protein alpha-q | 4 | 1 | HMG-CoA reductase | 3 | 3 |
| hGPCR33 | 4 | 3 | Orotidine 5'-phosphate decarboxylase | 3 | 2 |
| HPr(Ser) kinase/phosphorylase | 4 | 1 | Bromodomain-containing protein 2 | 2 | 1 |
| Lysis protein | 4 | 4 | Fucosyltransferase 5 | 2 | 1 |
| N-end-recognizing protein | 4 | 4 | Catenin beta-1 | 2 | 2 |
| N-WASP | 4 | 2 | Inhibitor of nuclear factor kappa B kinase alpha subunit | 2 | 2 |
| PriA helicase | 4 | 2 | Beta-galactosidase | 2 | 2 |
| Prohormone convertase | 4 | 4 | N(G),N(G)-dimethylarginine dimethylaminohydrolase 1 | 2 | 2 |
| RXR-interacting protein 14 | 4 | 2 | Vasopressin V1b receptor | 2 | 1 |
| Serotonin receptor 7 | 4 | 3 | 1-aminocyclopropane-1-carboxylate synthase 5 | 2 | 2 |
| S-mephenytoin 4-hydroxylase | 4 | 4 | D-alanine--D-alanine ligase | 2 | 2 |
| TrpRS | 4 | 1 | Serine/threonine-protein kinase RIO3 | 2 | 1 |
| UDPGDH | 4 | 4 | Steryl-sulfatase | 2 | 1 |
| UDP-GlcNAc-2-epimerase | 4 | 4 | Histone-lysine N-methyltransferase, H3 lysine-9 specific 3 | 2 | 2 |
| UDP-GlcNAc-2-epimerase | 4 | 4 | TRAF2- and NCK-interacting kinase | 2 | 1 |
| UDP-glucose 6-dehydrogenase | 4 | 4 | Neuropeptide FF receptor 1 | 2 | 2 |
| WASp | 4 | 2 | Squalene synthetase | 2 | 2 |
| None | 4 | 2 | Beta-lactamase type II | 2 | 2 |
| 3-deoxy-D-arabino-heptulosonate 7-phosphate synthase | 3 | 3 | Myosin light chain kinase family member 4 | 2 | 2 |
| 4,5-PCD | 3 | 3 | Beta-galactosidase | 2 | 2 |
| 5-HT1B | 3 | 1 | Human immunodeficiency virus type 1 reverse transcriptase | 2 | 1 |
| Non-enzyme | 3 | 3 | C-type lectin domain family 7 member A | 2 | 1 |
| Non-enzyme | 3 | 3 | Serine/threonine-protein kinase 17A | 2 | 1 |
| Non-enzyme | 3 | 3 | Vascular endothelial growth factor receptor 3 | 2 | 1 |
| Non-enzyme | 3 | 3 | Lysine-specific demethylase 2A | 2 | 2 |
| AHAS-II | 3 | 1 | Peptidyl-prolyl cis-trans isomerase D | 2 | 1 |
| AKIII | 3 | 3 | Mitogen-activated protein kinase kinase kinase 1 | 2 | 2 |
| Aspartate kinase | 3 | 3 | BMP-2-inducible protein kinase | 2 | 1 |
| Aspartate kinase 1 | 3 | 3 | Membrane-associated phosphatidylinositol transfer protein 1 | 2 | 1 |
| cGMP phosphodiesterase 6C | 3 | 1 | Serine/threonine-protein kinase SRPK3 | 2 | 1 |
| Cyclic nucleotide-binding protein | 3 | 3 | Neuropilin-1 | 2 | 2 |
| Cytochrome c oxidase subunit 1 | 3 | 3 | ATP-citrate synthase | 2 | 2 |
| Cytochrome c oxidase subunit 2 | 3 | 3 | Peptidyl-prolyl cis-trans isomerase NIMA-interacting 1 | 2 | 2 |
| Cytochrome c oxidase subunit 3 | 3 | 3 | Phospho-N-acetylmuramoyl-pentapeptide-transferase | 2 | 1 |
| Cytochrome c oxidase subunit 4 isoform 1, mitochondrial | 3 | 3 | Hematopoietic cell protein-tyrosine phosphatase 70Z-PEP | 2 | 2 |
| DHEA-ST | 3 | 3 | Epoxide hydratase | 2 | 2 |
| Diguanylate kinase | 3 | 3 | Anthrax lethal factor | 2 | 2 |
| Di-trans,poly-cis-decaprenylcistransferase | 3 | 2 | Aldehyde dehydrogenase 1A1 | 2 | 2 |
| Ecto-5'-nucleotidase | 3 | 2 | 6-phospho-1-fructokinase | 2 | 2 |
| eIF-4E | 3 | 2 | Histone acetyltransferase PCAF | 2 | 1 |
| Env polyprotein | 3 | 2 | Lysine-specific histone demethylase 1 | 2 | 1 |
| Glutamine phosphoribosylpyrophosphate amidotransferase | 3 | 2 | Dipeptidyl peptidase VIII | 2 | 2 |
| GNPDA 1 | 3 | 1 | Oxytocin receptor | 2 | 1 |
| GPATase | 3 | 2 | Tyrosine-protein kinase ABL2 | 2 | 1 |
| IMP--aspartate ligase | 3 | 3 | Chitinase | 2 | 2 |
| Inflammation-related G-protein coupled receptor EX33 | 3 | 3 | FkbO | 2 | 2 |
| Kinesin-related protein CENPE | 3 | 3 | Urokinase-type plasminogen activator | 2 | 2 |
| M-calpain | 3 | 3 | 5-enolpyruvylshikimate-3-phosphate synthase | 2 | 2 |
| Microsomal endopeptidase | 3 | 2 | Phosphatidylinositol-4-phosphate 5-kinase type-1 alpha | 2 | 1 |
| Mitoxantrone resistance-associated protein | 3 | 2 | D-alanylalanine synthetase | 2 | 2 |
| NS3 protein | 3 | 3 | Papain | 2 | 1 |
| Orphan nuclear receptor PXR | 3 | 3 | Serine/threonine-protein kinase MST2 | 2 | 1 |
| PelD | 3 | 3 | Low affinity neurotrophin receptor p75NTR | 1 | 1 |
| PPIase FKBP1A | 3 | 1 | Metabotropic glutamate receptor 5 | 1 | 1 |
| Receptor-interacting protein 1 | 3 | 3 | 3 beta-hydroxysteroid dehydrogenase/Delta 5-->4-isomerase | 1 | 1 |
| Serpin E1 | 3 | 3 | Glutathione S-transferase Mu 1 | 1 | 1 |
| SPCA | 3 | 3 | Interleukin-1 receptor-associated kinase 3 | 1 | 1 |
| Srr | 3 | 2 | DNA polymerase kappa | 1 | 1 |
| Steroid Delta-isomerase | 3 | 3 | Serine/threonine-protein kinase 10 | 1 | 1 |
| Transmembrane protein 142A | 3 | 3 | Alpha-galactosidase | 1 | 1 |
| TRPO | 3 | 3 | Histone acetyltransferase p300 | 1 | 1 |
| Tryptamin 2,3-dioxygenase | 3 | 3 | FK506 binding protein 12 | 1 | 1 |
| Tryptase II | 3 | 3 | Protein-tyrosine phosphatase 2C | 1 | 1 |
| Tryptophan oxygenase | 3 | 3 | Beta-lactamase | 1 | 1 |
| Voltage-gated calcium channel subunit alpha Cav1.2 | 3 | 3 | Heat shock protein 75 kDa, mitochondrial | 1 | 1 |
| None | 3 | 2 | Guanine deaminase | 1 | 1 |
| None | 3 | 2 | Aldehyde dehydrogenase dimeric NADP-preferring | 1 | 1 |
| 1,4-alpha-D-glucan glucanohydrolase | 2 | 2 | Phenylethanolamine N-methyltransferase | 1 | 1 |
| 11R-lipoxygenase | 2 | 2 | N-lysine methyltransferase SMYD2 | 1 | 1 |
| 2'-5'-oligoisoadenylate synthetase 1 | 2 | 1 | Mixed lineage kinase 7 | 1 | 1 |
| 2-oxoglutarate dehydrogenase, mitochondrial | 2 | 2 | Alpha-glucosidase MAL62 | 1 | 1 |
| 6-phosphofructokinase II | 2 | 2 | Methylenetetrahydrofolate dehydrogenase | 1 | 1 |
| Non-enzyme | 2 | 1 | Aminoacyl-tRNA synthetase | 1 | 1 |
| Non-enzyme | 2 | 2 | DNA polymerase beta | 1 | 1 |
| Non-enzyme | 2 | 2 | Kelch-like ECH-associated protein 1 | 1 | 1 |
| Abelson murine leukemia viral oncogene homolog 2 | 2 | 2 | IGF-like family receptor 1 | 1 | 1 |
| Adenylyl cyclase | 2 | 2 | Sphingosine 1-phosphate receptor Edg-1 | 1 | 1 |
| Adenylyl cyclase | 2 | 2 | Lymphocyte differentiation antigen CD38 | 1 | 1 |
| ALDH class 2 | 2 | 1 | Cytochrome P450 11A1 | 1 | 1 |
| Allantoate deiminase | 2 | 2 | Mitogen-activated protein kinase 15 | 1 | 1 |
| Alpha-glucan phosphorylase | 2 | 2 | Mandelate racemase | 1 | 1 |
| Amino acid biosynthesis regulatory protein | 2 | 2 | Myosin light chain kinase | 1 | 1 |
| Anthranilate synthase component I | 2 | 1 | Orotidine phosphate decarboxylase | 1 | 1 |
| Aspartate beta-decarboxylase | 2 | 2 | 3-oxoacyl-acyl-carrier protein reductase | 1 | 1 |
| Aspartokinase I/homoserine dehydrogenase I | 2 | 1 | Squalene synthase | 1 | 1 |
| AtCM1 | 2 | 2 | Mitogen-activated protein kinase kinase kinase 7 | 1 | 1 |
| ATP-binding cassette sub-family B member 1 | 2 | 2 | Hormonally up-regulated neu tumor-associated kinase | 1 | 1 |
| ATP-binding cassette, sub-family B, member 1 | 2 | 2 | Protein farnesyltransferase | 1 | 1 |
| ATP-dependent protease La | 2 | 1 | Enterobactin synthetase component E | 1 | 1 |
| Beta-galactoside-binding lectin L-14-I | 2 | 2 | Dual-specificity tyrosine-phosphorylation regulated kinase 2 | 1 | 1 |
| CFTR | 2 | 2 | Histone acetyltransferase KAT5 | 1 | 1 |
| Chitin synthase 1 | 2 | 2 | Peptide N-myristoyltransferase 1 | 1 | 1 |
| Chitin synthase 2 | 2 | 2 | Cyclin-dependent kinase-like 1 | 1 | 1 |
| Chitin-UDP acetyl-glucosaminyl transferase 3 | 2 | 2 | Serine/threonine-protein kinase NEK2 | 1 | 1 |
| Chloroplastic | 2 | 2 | Tyrosine-protein phosphatase non-receptor type 9 | 1 | 1 |
| Cholesterol acyltransferase 1 | 2 | 2 | Serine/threonine-protein kinase RIO2 | 1 | 1 |
| Chymotrypsin-C | 2 | 1 | Proline racemase | 1 | 1 |
| CM | 2 | 2 | Telomerase reverse transcriptase | 1 | 1 |
| DHOase | 2 | 2 | Transient receptor potential cation channel subfamily M member 8 | 1 | 1 |
| Dihydroorotase | 2 | 2 | Interferon-induced, double-stranded RNA-activated protein kinase | 1 | 1 |
| DNA helicase Rep and single-stranded DNA-dependent ATPase | 2 | 1 | Bromodomain-containing protein 4 | 1 | 1 |
| DNA-binding protein VF1 | 2 | 2 | Adaptor-associated kinase | 1 | 1 |
| Dopamine D4 receptor | 2 | 2 | 4-hydroxyphenylpyruvate dioxygenase | 1 | 1 |
| EF-G | 2 | 1 | Casein kinase II beta | 1 | 1 |
| FimX | 2 | 2 | Protein-tyrosine phosphatase LC-PTP | 1 | 1 |
| Glycine hydroxymethyltransferase | 2 | 2 | Interleukin-2 receptor alpha chain | 1 | 1 |
| GMD | 2 | 2 | P-hydroxybenzoate hydroxylase | 1 | 1 |
| Guanase | 2 | 2 | 6-phosphofructo-2-kinase/fructose-2,6-bisphosphatase 3 | 1 | 1 |
| HD domain protein | 2 | 2 | Orotidine phosphate decarboxylase | 1 | 1 |
| HDH | 2 | 1 | Citron Rho-interacting kinase | 1 | 1 |
| Hexokinase I | 2 | 2 | Beta-lactamase OXA-10 | 1 | 1 |
| Hexosephosphate aminotransferase | 2 | 2 | Thymidine kinase, mitochondrial | 1 | 1 |
| hPanK1 | 2 | 2 | Thymidine phosphorylase | 1 | 1 |
| ICD1 | 2 | 1 | Excitatory amino acid transporter 2 | 1 | 1 |
| Influenza A M2 channel | 2 | 2 | Glucose-6-phosphate translocase | 1 | 1 |
| K-sam | 2 | 2 | Serine-protein kinase ATR | 1 | 1 |
| Lactase | 2 | 2 | Serine/threonine-protein kinase MAK | 1 | 1 |
| Methionyl-tRNA synthetase | 2 | 2 | Dual specificity protein kinase CLK3 | 1 | 1 |
| Methionyl-tRNA synthetase | 2 | 2 | Protein VAC14 homolog | 1 | 1 |
| MGS | 2 | 2 | Baculoviral IAP repeat-containing protein 7 | 1 | 1 |
| Myosin heavy chain 7 | 2 | 1 | Alpha-galactosidase A | 1 | 1 |
| N-acetylglutamate kinase / N-acetylglutamate synthase | 2 | 2 | Eukaryotic translation initiation factor 2-alpha kinase 4 | 1 | 1 |
| NADH:nitrate reductase | 2 | 1 | 1-phosphatidylinositol 3-phosphate 5-kinase | 1 | 1 |
| NAGS | 2 | 2 | Aryl hydrocarbon receptor | 1 | 1 |
| nPKC-delta | 2 | 2 | Tyrosine- and threonine-specific cdc2-inhibitory kinase | 1 | 1 |
| Oleoyl-[acyl-carrier-protein] hydrolase | 2 | 2 | Carnitine palmitoyltransferase 2 | 1 | 1 |
| Oligomycin sensitivity conferral protein | 2 | 2 | Hepatitis C virus serine protease, NS3/NS4A | 1 | 1 |
| Oxoglutarate dehydrogenase (Succinyl-transferring), E1 component | 2 | 2 | NAD-dependent deacetylase sirtuin 1 | 1 | 1 |
| P26 | 2 | 2 | Glutamate dehydrogenase | 1 | 1 |
| p72-Syk | 2 | 2 | Bombesin receptor subtype-3 | 1 | 1 |
| Parkingson disease protein 7 | 2 | 1 | Histone-lysine N-methyltransferase, H3 lysine-9 specific 5 | 1 | 1 |
| PDT | 2 | 2 | Thymidylate kinase | 1 | 1 |
| PDT | 2 | 2 | Serine/threonine-protein kinase LATS1 | 1 | 1 |
| PGE2 receptor EP4 subtype | 2 | 1 | Bifunctional protein NCOAT | 1 | 1 |
| PHF-tau | 2 | 2 | STE20-like serine/threonine-protein kinase | 1 | 1 |
| Phosphopentokinase 1 | 2 | 2 | ATP-dependent RNA helicase DDX3X | 1 | 1 |
| Phosphorylase b kinase regulatory subunit alpha, skeletal muscle isoform | 2 | 2 | Lanosterol synthase | 1 | 1 |
| Phosphorylase kinase beta-subunit | 2 | 2 | S-methylmethionine--homocysteine S-methyltransferase BHMT2 | 1 | 1 |
| Phosphorylase kinase subunit gamma 1 | 2 | 2 | Homeodomain-interacting protein kinase 4 | 1 | 1 |
| PKC-A | 2 | 2 | Histo-blood group ABO system transferase | 1 | 1 |
| PNPase | 2 | 2 | Sulfate anion transporter 1 | 1 | 1 |
| Prephenate dehydratase | 2 | 2 | Beta-xylosidase | 1 | 1 |
| Prephenate dehydrogenase | 2 | 2 | Pregnane X receptor | 1 | 1 |
| Protein IPGM-1, isoform a | 2 | 2 | O-acetylserine sulfhydrylase | 1 | 1 |
| Putative uncharacterized protein PH0207 | 2 | 2 | N-arachidonyl glycine receptor | 1 | 1 |
| RAR-epsilon | 2 | 2 | Lactase-glycosylceramidase | 1 | 1 |
| RBP | 2 | 1 | Solute carrier family 22 member 12 | 1 | 1 |
| Recessive suppressor of secretory defect | 2 | 2 | Mitogen-activated protein kinase 7 | 1 | 1 |
| Regulatory protein SIR2 | 2 | 2 | Histone deacetylase-like amidohydrolase | 1 | 1 |
| Rhodobacter sphaeroides 2.4.1 chromosome 1, complete sequence | 2 | 2 | Homeodomain-interacting protein kinase 3 | 1 | 1 |
| RNA Polymerase | 2 | 2 | P2X purinoceptor 4 | 1 | 1 |
| Rts protein | 2 | 2 | G-protein coupled receptor 35 | 1 | 1 |
| RTX | 2 | 2 | UDP-N-acetylmuramoylalanine--D-glutamate ligase | 1 | 1 |
| SH2 domain-containing inositol 5'-phosphatase 1 | 2 | 2 | Intestinal alkaline phosphatase | 1 | 1 |
| SK2 | 2 | 1 | Ubiquitin carboxyl-terminal hydrolase 5 | 1 | 1 |
| Spermidine n1-acetyltransferase | 2 | 1 | D-alanyl-D-alanine dipeptidase | 1 | 1 |
| S-Rnase | 2 | 1 | Tyrosyl-DNA phosphodiesterase 1 | 1 | 1 |
| Ste2p | 2 | 2 | Vesicular acetylcholine transporter | 1 | 1 |
| Sulfonylurea receptor 1 | 2 | 2 | Homeodomain-interacting protein kinase 1 | 1 | 1 |
| Toxin B | 2 | 2 | GABA-A receptor; anion channel | 1 | 1 |
| Transcortin | 2 | 1 | Beta-galactosidase | 1 | 1 |
| Type II IPP | 2 | 2 | Dihydroorotate dehydrogenase | 1 | 1 |
| UPRT | 2 | 2 | Serine hydroxymethyltransferase, cytosolic | 1 | 1 |
| UPRTase | 2 | 2 | Neuronal acetylcholine receptor protein alpha-7 subunit | 1 | 1 |
| UPRTase | 2 | 2 | Endoplasmin | 1 | 1 |
| Voltage-gated calcium channel subunit alpha Cav3.3 | 2 | 2 | Histidase | 1 | 1 |
| None | 2 | 2 | Nitric-oxide synthase, brain | 1 | 1 |
| None | 2 | 2 | Endo-beta-N-acetylglucosaminidase | 1 | 1 |
| None | 2 | 1 | Glucosamine--fructose-6-phosphate aminotransferase [isomerizing] 1 | 1 | 1 |
| None | 2 | 2 | Glutamate [NMDA] receptor subunit epsilon 3 | 1 | 1 |
| None | 2 | 2 | EZH2/SUZ12/EED/RBBP7/RBBP4 | 1 | 1 |
| None | 2 | 2 | C-terminal processing protease of the D1 protein | 1 | 1 |
| (p)ppGpp synthase | 1 | 1 | Serine/threonine-protein kinase RIO1 | 1 | 1 |
| 1-phosphatidylinositol-4,5-bisphosphate phosphodiesterase gamma 1 | 1 | 1 | Fatty acid synthase | 1 | 1 |
| 2.7.11.1</ecNumber | 1 | 1 | Glutamine synthetase | 1 | 1 |
| 3-hydroxybutyrate dehydrogenase | 1 | 1 |  |  |  |
| 5'-phosphoribosylglycinamide transformylase | 1 | 1 |  |  |  |
| 67 kDa protein | 1 | 1 |  |  |  |
| 6-HDNO | 1 | 1 |  |  |  |
| Non-enzyme | 1 | 1 |  |  |  |
| Non-enzyme | 1 | 1 |  |  |  |
| Non-enzyme | 1 | 1 |  |  |  |
| 76 kDa lysosomal alpha-glucosidase | 1 | 1 |  |  |  |
| ACAT-2 | 1 | 1 |  |  |  |
| Acetohydroxy-acid synthase II small subunit | 1 | 1 |  |  |  |
| Acetokinase | 1 | 1 |  |  |  |
| ACR-20, isoform a | 1 | 1 |  |  |  |
| Adenylate nucleosidase | 1 | 1 |  |  |  |
| ADP-ribosylation factors guanine nucleotide-exchange protein 100 | 1 | 1 |  |  |  |
| AGK | 1 | 1 |  |  |  |
| AHAS-I | 1 | 1 |  |  |  |
| Alpha-2CAR | 1 | 1 |  |  |  |
| Alpha-isopropylmalate synthase | 1 | 1 |  |  |  |
| Alpha-NaCH | 1 | 1 |  |  |  |
| ALS-III | 1 | 1 |  |  |  |
| Amplified in liver cancer protein 1 | 1 | 1 |  |  |  |
| Annexin V | 1 | 1 |  |  |  |
| Anthranilate phosphoribosyltransferase | 1 | 1 |  |  |  |
| AP-TNAP | 1 | 1 |  |  |  |
| ARF1-directed GTPase-activating protein | 1 | 1 |  |  |  |
| Aurone synthase | 1 | 1 |  |  |  |
| AvrA | 1 | 1 |  |  |  |
| Beta-chimerin | 1 | 1 |  |  |  |
| Bifunctional phenylalanine ammonia-lyase | 1 | 1 |  |  |  |
| Brain-liver-intestine amiloride-sensitive Na(+) channel | 1 | 1 |  |  |  |
| C5a-R | 1 | 1 |  |  |  |
| cAMP and cGMP phosphodiesterase 11A | 1 | 1 |  |  |  |
| Carbon catabolite protein A | 1 | 1 |  |  |  |
| Caspase-5 subunit p10 | 1 | 1 |  |  |  |
| Catechol oxidase | 1 | 1 |  |  |  |
| Cav3.1c | 1 | 1 |  |  |  |
| Chaperone protein MSI3 | 1 | 1 |  |  |  |
| CKI-alpha | 1 | 1 |  |  |  |
| Coagulation factor IXa light chain | 1 | 1 |  |  |  |
| Complement C3b alpha' chain | 1 | 1 |  |  |  |
| Complex III subunit III | 1 | 1 |  |  |  |
| CooA protein | 1 | 1 |  |  |  |
| Copper-sensitive operon repressor | 1 | 1 |  |  |  |
| CPSase I | 1 | 1 |  |  |  |
| CTP synthetase | 1 | 1 |  |  |  |
| CXCR-7 | 1 | 1 |  |  |  |
| Cyclophilin A | 1 | 1 |  |  |  |
| Cytosolic IMP-GMP specific 5'-nucleotidase | 1 | 1 |  |  |  |
| D-alanine:D-alanine ligase | 1 | 1 |  |  |  |
| Deadenylating nuclease | 1 | 1 |  |  |  |
| Dimeric hemoglobin | 1 | 1 |  |  |  |
| Dipeptidyl peptidase-like protein 9 | 1 | 1 |  |  |  |
| D-methionine transport system permease protein metI | 1 | 1 |  |  |  |
| DNA topoisomerase II | 1 | 1 |  |  |  |
| E3 ubiquitin ligase complex SCF subunit CDC4 | 1 | 1 |  |  |  |
| Ecto-NAD+ glycohydrolase | 1 | 1 |  |  |  |
| ELIC | 1 | 1 |  |  |  |
| G6PD | 1 | 1 |  |  |  |
| Galactose operon repressor | 1 | 1 |  |  |  |
| Galectin | 1 | 1 |  |  |  |
| GALR-2 | 1 | 1 |  |  |  |
| Gamma-ENaC | 1 | 1 |  |  |  |
| Gamma-glutamate kinase | 1 | 1 |  |  |  |
| Gibberellin-insensitive dwarf protein 1 | 1 | 1 |  |  |  |
| Glutamate carboxypeptidase-like protein 2 | 1 | 1 |  |  |  |
| Glutamine amido-transferase | 1 | 1 |  |  |  |
| Glutamine amidotransferase:cyclase | 1 | 1 |  |  |  |
| Glycerokinase | 1 | 1 |  |  |  |
| Glycerol dehydrase beta subunit | 1 | 1 |  |  |  |
| Glycerol dehydrase gamma subunit | 1 | 1 |  |  |  |
| Glycerol dehydratase large subunit | 1 | 1 |  |  |  |
| GMP-PDE gamma | 1 | 1 |  |  |  |
| GPD-C | 1 | 1 |  |  |  |
| GSH-S | 1 | 1 |  |  |  |
| GST class-pi | 1 | 1 |  |  |  |
| HBP23 | 1 | 1 |  |  |  |
| HD4 | 1 | 1 |  |  |  |
| HDC | 1 | 1 |  |  |  |
| Heat shock protein HslU | 1 | 1 |  |  |  |
| HGF activator | 1 | 1 |  |  |  |
| HL-60 PAD | 1 | 1 |  |  |  |
| HM63 | 1 | 1 |  |  |  |
| HMG-CoA reductase | 1 | 1 |  |  |  |
| hMSH2 | 1 | 1 |  |  |  |
| hPanK2 | 1 | 1 |  |  |  |
| hPanK3 | 1 | 1 |  |  |  |
| HPr kinase/phosphatase | 1 | 1 |  |  |  |
| HSCARG | 1 | 1 |  |  |  |
| Hypothetical phosphoserine phosphatase | 1 | 1 |  |  |  |
| IDH kinase/phosphatase | 1 | 1 |  |  |  |
| IL-2 | 1 | 1 |  |  |  |
| ImGP synthase subunit hisF | 1 | 1 |  |  |  |
| Intracellular protease I | 1 | 1 |  |  |  |
| IRK-1 | 1 | 1 |  |  |  |
| Iron(III) dicitrate transport protein fecA | 1 | 1 |  |  |  |
| Isopropylmalate/homocitrate/citramalate synthase | 1 | 1 |  |  |  |
| Kinesin-related protein HSET | 1 | 1 |  |  |  |
| Lasalocid biosynthesis protein Lsd19 | 1 | 1 |  |  |  |
| L-asparaginase I | 1 | 1 |  |  |  |
| LivG | 1 | 1 |  |  |  |
| L-LDH | 1 | 1 |  |  |  |
| L-LDH 2 | 1 | 1 |  |  |  |
| L-serine dehydratase (Iron, sulfur-dependent) | 1 | 1 |  |  |  |
| M.EcoDam | 1 | 1 |  |  |  |
| MALT lymphoma-associated translocation | 1 | 1 |  |  |  |
| Metacaspase MCA2 | 1 | 1 |  |  |  |
| MetN | 1 | 1 |  |  |  |
| MHC class II antigen DRA | 1 | 1 |  |  |  |
| MHCK-A | 1 | 1 |  |  |  |
| Mitochondrial uncoupling protein | 1 | 1 |  |  |  |
| Mutated in multiple advanced cancers 1 | 1 | 1 |  |  |  |
| MutT/nudix family protein | 1 | 1 |  |  |  |
| Myeloproliferative leukemia protein | 1 | 1 |  |  |  |
| Myosin-Va | 1 | 1 |  |  |  |
| Na(+)/Ca(2+)-exchange protein 1 | 1 | 1 |  |  |  |
| Na(+)/PI cotransporter 1 | 1 | 1 |  |  |  |
| N-acetyl-L-glutamate 5-phosphotransferase | 1 | 1 |  |  |  |
| NAD(P)(+) transhydrogenase [B-specific] | 1 | 1 |  |  |  |
| NAGSA dehydrogenase | 1 | 1 |  |  |  |
| Nuclear matrix protein 265 | 1 | 1 |  |  |  |
| Nuclear receptor subfamily 1 group A member 1 | 1 | 1 |  |  |  |
| OAS-TL A | 1 | 1 |  |  |  |
| Oligosaccharyltransferase | 1 | 1 |  |  |  |
| OTRPC1 | 1 | 1 |  |  |  |
| PDT | 1 | 1 |  |  |  |
| P-element transposase | 1 | 1 |  |  |  |
| Phospholipase A2 | 1 | 1 |  |  |  |
| PI3Kalpha | 1 | 1 |  |  |  |
| PilZ domain protein | 1 | 1 |  |  |  |
| Platelet membrane glycoprotein IIb | 1 | 1 |  |  |  |
| pMMO-H alpha subunit | 1 | 1 |  |  |  |
| Protein Dhm1 | 1 | 1 |  |  |  |
| Psi-conotoxin P3.8 | 1 | 1 |  |  |  |
| PTE | 1 | 1 |  |  |  |
| Pyrophosphate-dependent 6-phosphofructose-1-kinase | 1 | 1 |  |  |  |
| Pyrophosphate--fructose 6-phosphate 1-phosphotransferase subunit beta | 1 | 1 |  |  |  |
| QAPRTase | 1 | 1 |  |  |  |
| Rab geranylgeranyl transferase componenet, subunit beta | 1 | 1 |  |  |  |
| Rab GG transferase alpha | 1 | 1 |  |  |  |
| RecA, E. coli, homolog of | 1 | 1 |  |  |  |
| Regulatory protein SIR2 homolog 2 | 1 | 1 |  |  |  |
| RuvB Protein | 1 | 1 |  |  |  |
| SAPKK1 | 1 | 1 |  |  |  |
| Selenocysteine lyase | 1 | 1 |  |  |  |
| Shaw2 | 1 | 1 |  |  |  |
| STE20-like kinase MST | 1 | 1 |  |  |  |
| StyR | 1 | 1 |  |  |  |
| T4-binding globulin | 1 | 1 |  |  |  |
| Teichoic acid biosynthesis protein D | 1 | 1 |  |  |  |
| Transcriptional regulator, MarR family | 1 | 1 |  |  |  |
| Troponin I, cardiac muscle | 1 | 1 |  |  |  |
| Tryptophan RNA-binding attenuator protein | 1 | 1 |  |  |  |
| Ubiquitin-conjugating enzyme E2-CDC34 | 1 | 1 |  |  |  |
| Uncharacterized protein | 1 | 1 |  |  |  |
| Uncharacterized protein | 1 | 1 |  |  |  |
| VAChT | 1 | 1 |  |  |  |
| None | 1 | 1 |  |  |  |
| None | 1 | 1 |  |  |  |
| None | 1 | 1 |  |  |  |
| None | 1 | 1 |  |  |  |
