# Supplementary material for: Are there physicochemical differences between allosteric and competitive ligands?
Source: PLoS Comput Biol. 2017 Nov 10;13(11):e1005813. doi: 10.1371/journal.pcbi.1005813 (PMC5699844; doi:10.1371/journal.pcbi.1005813)
Supplement: S3 Table — Numbers in bold denote differences between allosteric and competitive compounds with p<0.0001 and no overlap in 95%ci of medians. This analysis is done with the centers of the protein-ligand clusters. (DOCX) [file pcbi.1005813.s005.docx]

**Table S3.** Medians (95%ci) of the 29 physicochemical properties. Numbers in bold denote differences between allosteric and competitive compounds with p<0.0001 and no overlap in 95%ci of medians. **This analysis is done with the centers of the protein-ligand clusters.**

|  | **60%/0.6** | | **75%/0.75** | | **90%/0.9** | | **100%/1.0** | |
| --- | --- | --- | --- | --- | --- | --- | --- | --- |
| **Properties** | Allosteric | Competitive | Allosteric | Competitive | Allosteric | Competitive | Allosteric | Competitive |
| a_heavy | 25 (±0) | 26 (±1) | **26 (±0)** | **27 (±0)** | 28 (±0) | 28 (±1) | **28 (±0)** | **29 (±0)** |
| a_aro | 12 (±0) | 12 (±0) | 12 (±0) | 12 (±0) | **15 (±0)** | **12 (±0)** | **15 (±0)** | **12 (±0)** |
| **a_aro/HA** | **0.50 (±0.01)** | **0.45 (±0.01)** | **0.50 (±0.01)** | **0.462 (±0.007)** | **0.515 (<0.001)** | **0.444 (±0.007)** | **0.51 (±0.01)** | **0.429 (±0.006)** |
| a_acc | **3 (±0)** | **4 (±0)** | 4 (±0) | 4 (±0) | 4 (±0) | 4 (±0) | 4 (±0) | 4 (±0) |
| **a_acc/HA** | **0.136 (<0.001)** | **0.148 (±0.005)** | **0.136 (±0.002)** | **0.143 (<0.001)** | **0.138 (<0.001)** | **0.143 (<0.001)** | **0.136 (<0.001)** | **0.143 (±0.004)** |
| **a_don** | **1 (±0)** | **2 (±0)** | **1 (±0)** | **2 (±0)** | **1 (±0)** | **2 (±0)** | **1 (±0)** | **2 (±0)** |
| **a_don/HA** | **0.045 (<0.001)** | **0.083 (±0.003)** | **0.042 (<0.001)** | **0.077 (±0.003)** | **0.038 (<0.001)** | **0.074 (±0.003)** | **0.037 (<0.001)** | **0.076 (±0.002)** |
| a_acid | 0 (±0) | 0 (±0) | 0 (±0) | 0 (±0) | 0 (±0) | 0 (±0) | 0 (±0) | 0 (±0) |
| a_acid/HA | 0 (±0) | 0 (±0) | 0 (±0) | 0 (±0) | 0 (±0) | 0 (±0) | 0 (±0) | 0 (±0) |
| a_base | 0 (±0) | 0 (±0) | 0 (±0) | 0 (±0) | 0 (±0) | 0 (±0) | 0 (±0) | 0 (±0) |
| a_base/HA | 0 (±0) | 0 (±0) | 0 (±0) | 0 (±0) | 0 (±0) | 0 (±0) | 0 (±0) | 0 (±0) |
| **b_count** | **45 (±1)** | **49 (±1)** | **47 (±0)** | **52 (±1)** | **51 (±1)** | **55 (±1)** | **51 (±0)** | **56 (±0)** |
| **b_count/HA** | **1.806 (±0.006)** | **1.91 (±0.01)** | **1.800 (<0.001)** | **1.930 (±0.008)** | **1.804 (±0.004)** | **1.943 (±0.005)** | **1.812 (±0.002)** | **1.951 (±0.005)** |
| b_ar | 12 (±0) | 12 (±0) | 12 (±0) | 12 (±0) | **16 (±0)** | **12 (±0)** | **16 (±0)** | **12 (±0)** |
| **b_ar/HA** | **0.5 (±0)** | **0.461 (±0.007)** | **0.515 (±0.002)** | **0.462 (±0.009)** | **0.522 (±0.006)** | **0.444 (±0.008)** | **0.515 (<0.001)** | **0.43 (±0.01)** |
| **b_1rotN** | **4 (±0)** | **5 (±0)** | **4 (±0)** | **5 (±0)** | **5 (±0)** | **6 (±0)** | **5 (±0)** | **6 (±0)** |
| **b_1rotN/HA** | **0.167 (<0.001)** | **0.185 (±0.003)** | **0.167 (<0.001)** | **0.188 (±0.002)** | **0.172 (<0.001)** | **0.195 (±0.005)** | **0.174 (<0.001)** | **0.2 (±0)** |
| FCharge | 0 (±0) | 0 (±0) | 0 (±0) | 0 (±0) | 0 (±0) | 0 (±0) | 0 (±0) | 0 (±0) |
| FCharge/HA | 0 (±0) | 0 (±0) | 0 (±0) | 0 (±0) | 0 (±0) | 0 (±0) | 0 (±0) | 0 (±0) |
| **SlogP** | **3.44 (±0.03)** | **3.27 (±0.07)** | **3.61 (±0.02)** | **3.53 (±0.04)** | **3.85 (±0.01)** | **3.63 (±0.03)** | **3.89 (±0.01)** | **3.59 (±0.03)** |
| **a_nC/HA** | **0.731 (±0.003)** | **0.741 (±0.004)** | **0.731 (±0.001)** | **0.750 (±0.006)** | **0.731 (±0.001)** | **0.750 (±0.006)** | **0.731 (±0.001)** | **0.744 (±0.006)** |
| logS | -4.50 (±0.03) | -4.62 (±0.09) | **-4.80 (±0.02)** | **-4.89 (±0.07)** | -5.13 (±0.01) | -5.13 (±0.04) | -5.15 (±0.01) | -5.15 (±0.04) |
| chiral | 0 (±0) | 0 (±1) | **0 (±0)** | **1 (±0)** | **0 (±0)** | **1 (±0)** | **0 (±0)** | **1 (±0)** |
| chiral/HA | 0 (±0) | 0.00 (±0.03) | **0 (±0)** | **0.029 (±0.002)** | **0 (±0)** | **0.032 (±0.001)** | **0 (±0)** | **0.033 (±0.001)** |
| rings | 3 (±0) | 3 (±0) | 3 (±0) | 3 (±0) | 4 (±0) | 3 (±1) | 4 (±0) | 3 (±1) |
| lip_druglike | 1 (±0) | 1 (±0) | 1 (±0) | 1 (±0) | 1 (±0) | 1 (±0) | 1 (±0) | 1 (±0) |
| lip_violation | 0 (±0) | 0 (±0) | 0 (±0) | 0 (±0) | 0 (±0) | 0 (±0) | 0 (±0) | 0 (±0) |
| opr_leadlike | 1 (±0) | 1 (±0) | 1 (±0) | 1 (±0) | 1 (±0) | 1 (±0) | 1 (±0) | 1 (±0) |
| opr_violation | 0 (±0) | 1 (±0) | **0 (±0)** | **1 (±0)** | 1 (±0) | 1 (±0) | 1 (±0) | 1 (±0) |
